# Omega-3 fatty acid supplementation improves skeletal muscle mitochondrial function in a model of Barth syndrome

**DOI:** 10.1172/jci.insight.196134

**Published:** 2026-06-09

**Authors:** Katharina B. Kuentzel, Ana Vranešević, Samuel A.J. Trammell, Fabian Finger, Jesper F. Havelund, Yvette L. Schooneveldt, Ivan Bradić, Nicoline R. Andersen, Anna S. Hassing, Katja T. Michler, Martin R. Larsen, Zachary Gerhart-Hines, Steven M. Claypool, Jonas T. Treebak, Andreas M. Fritzen, Matthew P. Gillum, Steen Larsen, Nils Færgeman, Trisha J. Grevengoed

**Affiliations:** 1 Department of Biomedical Sciences, and; 2 Novo Nordisk Foundation Center for Metabolic Research, University of Copenhagen, Copenhagen, Denmark.; 3 Center for Adipocyte Signaling (ADIPOSIGN), and; 4 Department of Biochemistry and Molecular Biology, University of Southern Denmark, Odense, Denmark.; 5 Johns Hopkins University School of Medicine, Baltimore, Maryland, USA.; 6 Institute of Sports Medicine Copenhagen, Department of Orthopedic Surgery M, Copenhagen University Hospital – Bispebjerg and Frederiksberg, Denmark.; 7 Clinical Research Centre, Medical University of Bialystok, Bialystok, Poland.

**Keywords:** Metabolism, Muscle biology, Lipidomics, Mitochondria

## Abstract

The composition of mitochondrial membrane lipids is crucial to cellular respiration, as seen in Barth syndrome (BTHS), a rare disease affecting skeletal muscle, heart, and neutrophils. In BTHS, mutations in the tafazzin (*TAZ*) gene reduce remodeling of the mitochondrial phospholipid cardiolipin, causing mitochondrial dysfunction in skeletal muscle and heart. Here, we investigated effects of altering polyunsaturated fatty acid content in cardiolipin using preclinical models of BTHS. In vitro, the absence of TAZ did not impair omega-3 fatty acid incorporation into cardiolipin and resulted in increased turnover of these acyl chains. To examine this in a functional model, we generated mice with muscle-specific knockout of *Taz* (*TAZ* *MKO* mice), which recapitulated the human phenotype in skeletal muscle. Supplementing the diet of *TAZ* *MKO* mice with fish oil–derived omega-3 fatty acids prevented lean mass loss, improved mitochondrial respiration, altered mitochondrial structure, and revealed moderate improvements in the stress response. Surprisingly, no diet-induced changes in cardiolipin species were observed in the *TAZ* *MKO* mice, but other phospholipids were altered by both genotype and diet, revealing complex regulation and potential compensation. Overall, this work provides evidence that omega-3 fatty acid supplementation is beneficial in muscle lacking TAZ to improve quality of life when added to current BTHS treatments.

## Abstract 2

Mitochondrial defects are improved by omega-3 fatty acid feeding in muscle of mice modelling Barth syndrome, a childhood cause of heart failure and muscle weakness

## Graphical abstract

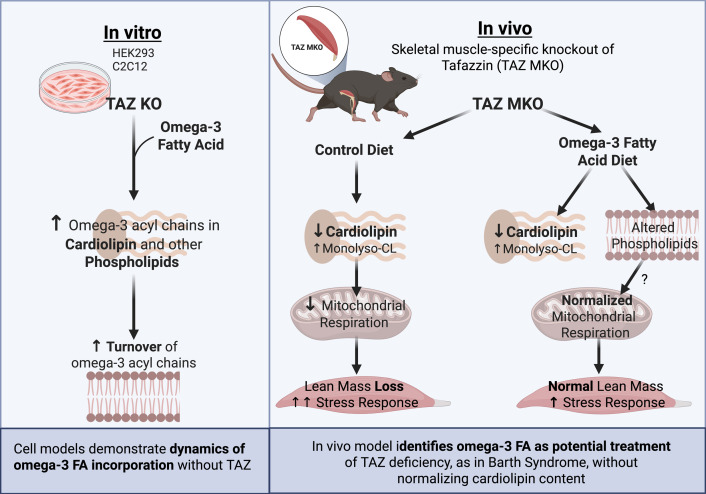

## Introduction

Barth syndrome (BTHS) is a rare, life-threatening X-linked disease that causes skeletal muscle weakness, neutropenia, and heart failure in children under 3 years of age (1). The disease is caused by a mutation in the tafazzin (*TAZ*) gene, and lack of functional TAZ protein prevents remodeling of the mitochondrial phospholipid, cardiolipin (CL) (2). CL is necessary to form the inner mitochondrial membrane and the normal structure of cristae. Nascent CL is synthesized by CL synthase (3) from phosphatidylglycerol and cytidinephosphate-diacylglyerol and then extensively remodeled in heart and skeletal muscle, with the predominant species containing linoleic acid (LA, C18:2) at all 4 positions of this lipid dimer to form tetralinoleoyl-CL. In BTHS, TAZ is unable to remodel CL, leading to a decrease in mature CL (tetralinoleoyl-CL) and an increase in monolyso-CL (MLCL), the remodeling intermediate (4). The increase in MLCL and decrease in CL impairs formation of the inner mitochondrial membrane, thus altering cristae formation and impairing protein binding and stability (5, 6), which contribute to mitochondrial dysfunction, ultimately leading to impaired skeletal muscle and heart function (7). Gene therapy to replace the nonfunctional *TAZ* gene is in development (8–11), but additional strategies are urgently needed as current treatment primarily aims at symptom reduction. Likewise, fatigue and muscle weakness contribute to markedly impaired quality of life in boys and men with BTHS (12), indicating a major importance to improving skeletal muscle function in BTHS.

Omega-3 fatty acids are essential for development and play roles in many pathways, including increasing mitochondrial biogenesis and regulating inflammation (13–15). These fatty acids are a necessary component of membrane lipids, and their incorporation into phospholipids is generally proportional to their dietary intake, which tends to be low in most Western diets (14). The CL profile of a tissue can change with diet or other conditions to increase the amount of species containing docosahexaenoic acid (DHA, C22:6) (16–19). In aging or obesity, it may diminish mitochondrial function to have less LA and more DHA in CL (20), but diet-induced elevation of DHA is unlikely to impair function (21).

The importance of the acyl-chain composition of CL is an ongoing debate in finding treatments for BTHS and understanding more subtle changes with aging and obesity. The high prevalence of LA in skeletal muscle and heart CL suggests that this composition is beneficial in tissues of high oxidative capacity, although this is not necessary in yeast (22). Typical diets in Western civilization contain high amounts of LA, so it is unlikely that dietary deficiency of LA would contribute to the lack of remodeled CL containing this acyl chain. Indeed, supplementation of LA in the inducible TAZ-knockdown mice had little effect on CL or MLCL content (23). LA supplementation in utero only slightly altered cardiac CL and MLCL levels (24), and later onset of diet change failed to improve cardiomyopathy (25). Western diets are also more likely to be deficient in omega-3 fatty acids, such as DHA and eicosapentaenoic acid (EPA, C20:5), but the role of these fatty acids in CL function is unclear. Additionally, other phospholipids are altered with BTHS (26, 27) or by diet (28, 29), and these might have the ability to contribute to or compensate for the defects associated with loss of TAZ function. In BTHS patients, heart transplantation has not been shown to improve skeletal muscle function and exercise tolerance (30). Therefore, we explored the possibility that elevating omega-3 fatty acid intake through omega-3 fatty acid supplementation could improve outcomes in a skeletal muscle–specific model of TAZ deficiency, as this strategy could be a highly feasible addition to BTHS treatment.

## Results

### DHA is preferentially incorporated into CL in cells lacking TAZ.

**Figure 1:**
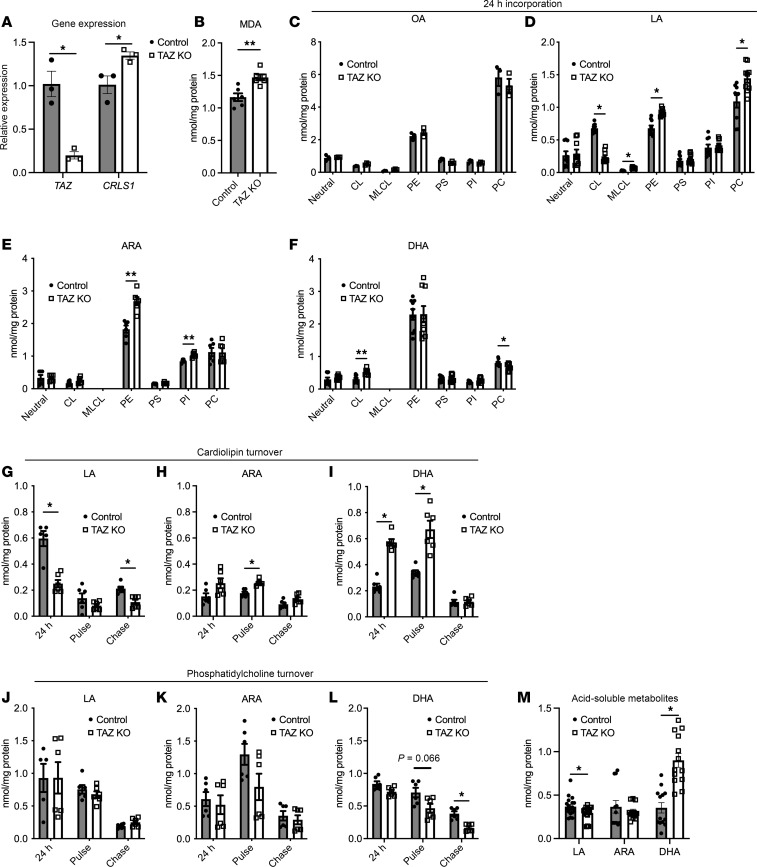
DHA is preferentially incorporated into CL in cells lacking TAZ. (**A**) Gene expression in HEK293 cells (*n* = 3). (**B**) Lipid peroxidation malondialdehyde (MDA) measured by TBARS assay (*n* = 6). (**C–F**) HEK293 cells were incubated with a mix of fatty acids and 1-[^14^C]–labeled fatty acid for 24 hours. Experiments were performed in triplicate in 2 or 3 independent assays. (**G**–**L**) HEK293 cells were incubated with a mix of fatty acids and 1-[^14^C]–labeled fatty acid for 24 hours, 4 hours (pulse), or 4 hours and then 20 hours with only unlabeled fatty acid (chase). Experiments were performed in triplicate in 2–3 independent assays. (**M**) Acid-soluble metabolites in media after 4 hours of incubation (pulse). Measurement was performed in 6 wells/experiment in 2–3 independent assays. Statistical significance was determined by unpaired Student’s *t* test (**B**) or multiple *t* tests with the Holm-Šídák method for multiple comparison testing (**A** and **C**–**M**) where appropriate. *P < 0.05; **P ≤ 0.01.

HEK293 cells lacking *TAZ* (31) displayed an increase in CL synthase (*CRLS1*) gene expression (Figure 1A) and malondialdehyde (MDA) levels (Figure 1B). Elevated lipid peroxidation upon TAZ deficiency might be associated with increased cellular stress (32, 33); thus, our cells resemble a valid in vitro model. Without TAZ, cells cannot remodel CL to incorporate LA, leading to higher MLCL and CL that contains less LA (4), but the extent to which other fatty acids are incorporated into CL to compensate for the reduced LA remains poorly understood. Thus, we investigated how different fatty acids are incorporated and turned over into phospholipids. We used a relatively physiologic mixture of saturated, mono-, and polyunsaturated fatty acids with a [^14^C]-labeled fatty acid tracer to follow its incorporation or breakdown. While the incorporation of oleic acid (OA, C18:1) was unchanged in *TAZ*-knockout (*TAZ* *KO*) cells (Figure 1C), the use of polyunsaturated fatty acids was altered (Figure 1, D–F). As anticipated (34), *TAZ* *KO* cells incorporated less LA into CL, but increased incorporation of other fatty acids, especially DHA (Figure 1, D–F), indicating a potential compensation with these fatty acids. Without TAZ, less DHA was incorporated into phosphatidylcholine (PC), a major donor of acyl chains for CL (Figure 1F), suggesting a shift in DHA utilization in TAZ-deficient cells. Additionally, we observed more MLCL with OA and LA in *TAZ* *KO* cells, but were unable to detect any MLCL with labeled arachidonic acid (ARA, C20:4) or DHA (Figure 1, C and E). Higher amounts of ARA were incorporated into phosphatidylethanolamine (PE) and phosphatidylinositol (PI) in *TAZ* *KO* cells (Figure 1E), showing how altered CL remodeling has a wide range of effects on the different phospholipids.

In a pulse-chase experiment, we observed that control cells had a time-dependent incorporation of LA, which could be consistent with LA being incorporated into one phospholipid before being transferred to CL (Figure 1G and Supplemental Figure 1, A–E; supplemental material available online with this article; https://doi.org/10.1172/jci.insight.196134DS1). In the *TAZ* *KO* cells, we observed decreased incorporation of LA into CL at 24 hours and after chase, the time points where this transacylase activity would be important (Figure 1G). The incorporation of [^14^C]DHA or [^14^C]ARA in CL was elevated in *TAZ* *KO* cells (Figure 1, H and I) after the pulse, but not after the chase, indicating a higher turnover. The highest turnover was observed with DHA, indicating that a steady supply of DHA is required to maintain the high DHA content in CL in *TAZ* *KO* cells. Corresponding levels of DHA in PC, a major acyl-chain donor for CL, were also lower in the *TAZ* *KO* cells, whereas the other fatty acids displayed less clear effects (Figure 1, J–L). Additionally, we observed larger release of partially degraded [^14^C]DHA into the media as acid-soluble metabolites from *TAZ* *KO* cells (Figure 1M). Higher oxidative stress and dysfunctional mitochondria (32) may contribute to turnover of the highly oxidizable DHA in TAZ-deficient cells, thus making a continuous supply of these molecules potentially beneficial.

### Muscle cells lacking TAZ incorporate DHA and EPA into CL.

Because BTHS strongly affects specific cell types, such as muscle, we tested whether knocking out TAZ in the myoblast-like cell line C2C12 would alter the incorporation of exogenously provided omega-3 fatty acids into CL. Differentiated WT and *TAZ* *KO* C2C12 cells were treated for 2 days with a mixture of DHA and EPA at a ratio similar to menhaden fish oil (FO). Of note, *TAZ* *KO* cells do not differentiate as well as the WT controls (35).

**Figure 2:**
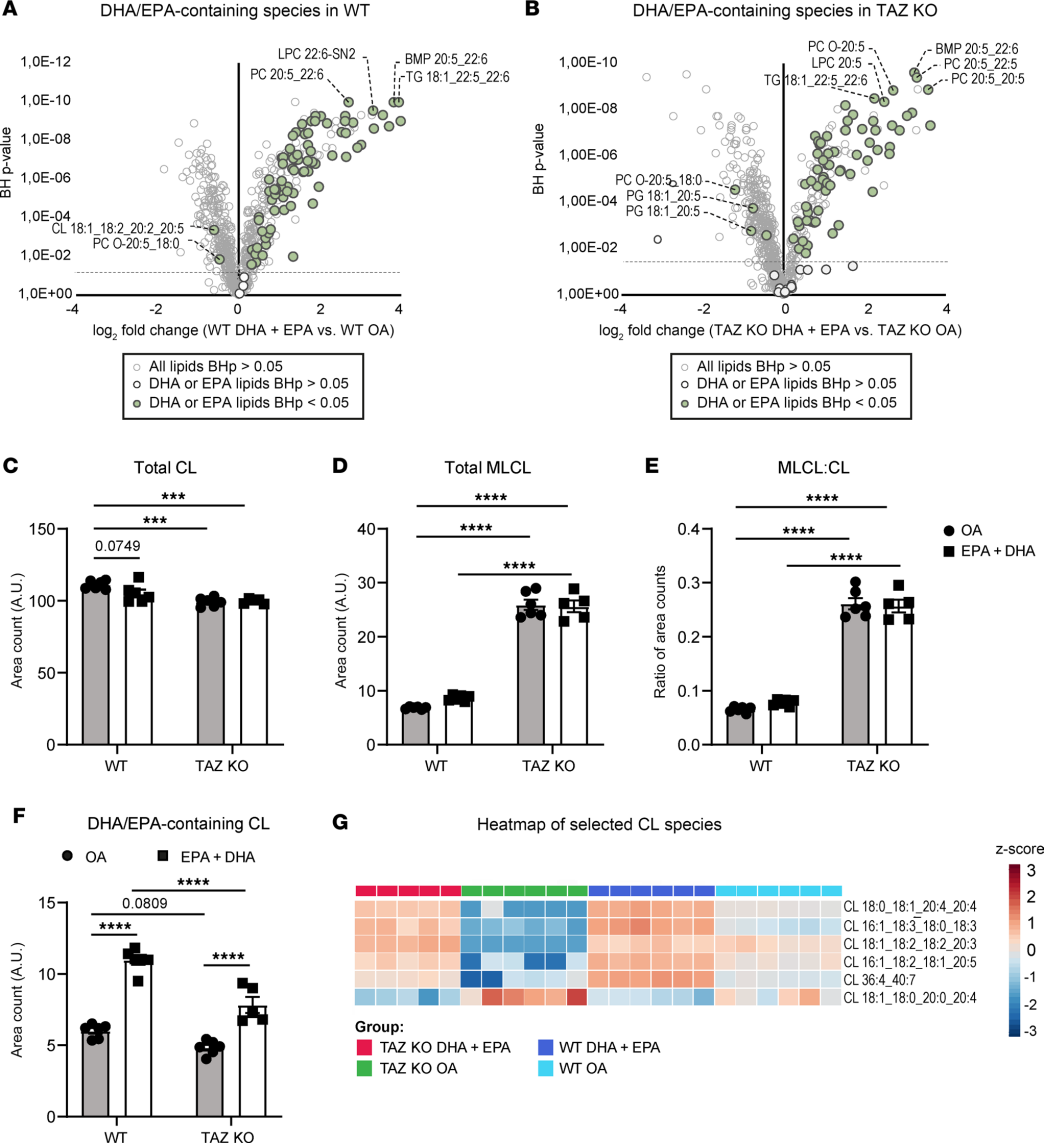
Muscle cells lacking TAZ incorporate DHA and EPA into CL. Volcano plots of DHA- and EPA-containing lipids in (**A**) WT and (**B**) *TAZ* *KO* C2C12 cells. (**C**) Total cardiolipin (CL). (**D**) Total monolyso-cardiolipin (MLCL). (**E**) MLCL-to-CL ratio. (**F**) All DHA- or EPA-containing CL species. (**G**) Heatmap of selected CL species. *n* = 5–6. Statistical significance was determined by 2-way ANOVA with the Holm-Šídák method for multiple comparison testing (**C**–**F**). ***P ≤ 0.001; ****P ≤ 0.0001.

DHA and EPA treatment led to high incorporation into CL of these fatty acids (Figure 2, A and B), as well as changes in various lipid classes (Supplemental Figure 2, A–D) in WT and *TAZ* *KO* cells. Genotype was a major driver of CL levels and species, with *TAZ* *KO* cells displaying reduced total CL and elevated MLCL (Figure 2, C–E). DHA and EPA treatment did not alter total CL or MLCL in the *TAZ* *KO* cells (Figure 2, C and D), but did change the CL species containing DHA or EPA (Figure 2F), and normalized several CL species containing EPA, LA, or ARA to WT levels (Figure 2G and Supplemental Figure 2E). Of note, *TAZ* *KO* cells incorporated less DHA and EPA into CL than WT controls (Figure 2F). Thus, providing DHA and EPA to muscle cells lacking TAZ leads to their incorporation into CL and may normalize some CL species.

### Mice lacking TAZ in skeletal muscle display impaired mitochondrial respiration and lower lean mass.

**Figure 3:**
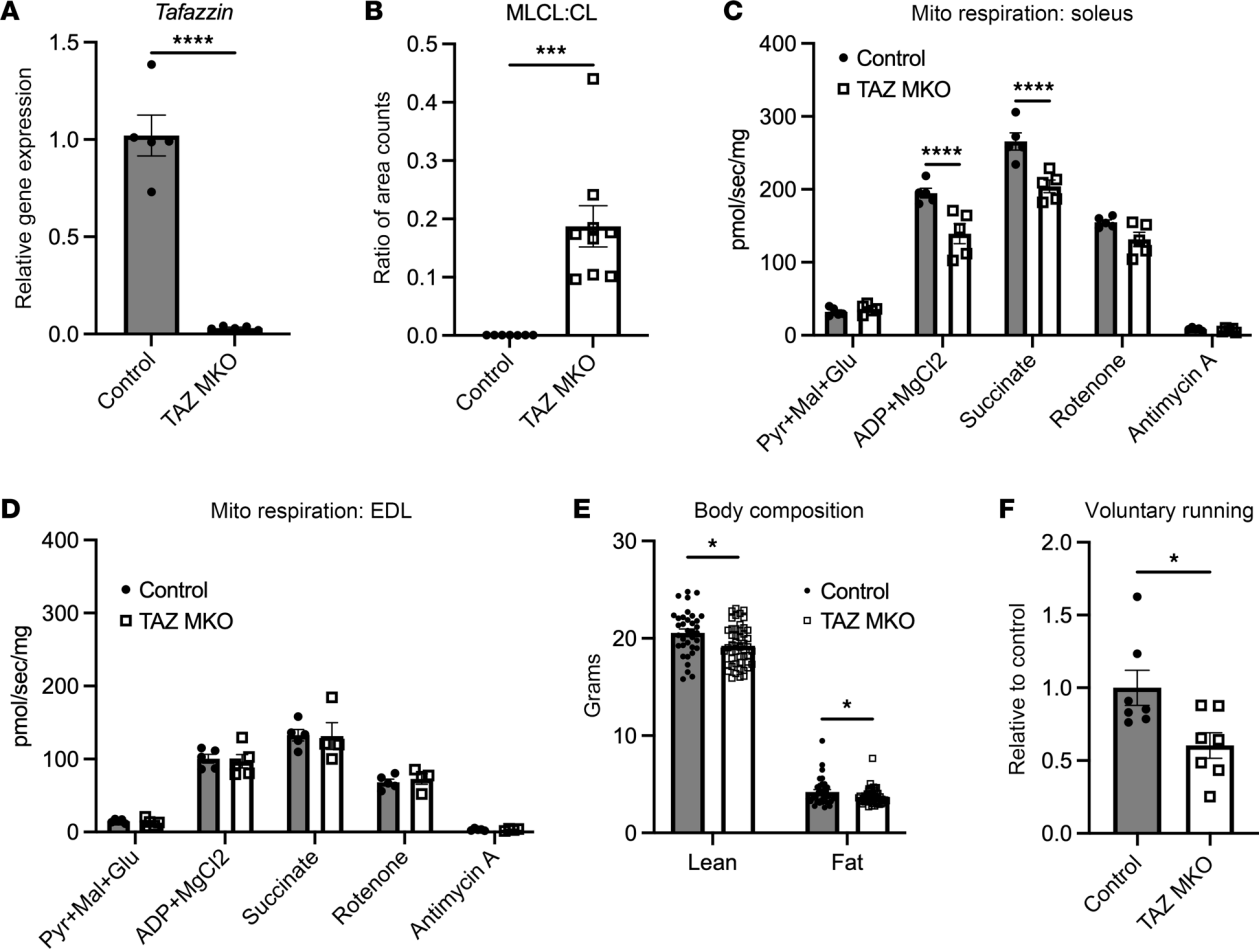
Mice lacking TAZ in skeletal muscle display impaired mitochondrial respiration and lower lean mass. (**A**) Gene expression in gastrocnemius (*n* = 5). (**B**) Ratio of total MLCL to total CL area (*n* = 7–9). (**C** and **D**) Mitochondrial respiration in soleus and EDL (*n* = 5). (**E**) Body composition by MRI in chow-fed mice (*n* = 35–48). (**F**) Voluntary wheel running (*n* = 7). Statistical significance was determined by *t* tests (**A**, **B**, and **F**) or multiple *t* tests (**C**–**E**) with the Holm-Šídák method for multiple comparison testing where appropriate. *P < 0.05; ***P ≤ 0.001; ****P ≤ 0.0001.

To determine whether the high DHA or EPA incorporation and turnover in TAZ-deficient cells (Figure 1I and Figure 2F) could be functionally relevant in vivo, we generated a skeletal muscle–specific *Taz*-KO mouse line (*Taz^loxP/Y^xHSA-cre*; *TAZ* *MKO*), using previously generated *Taz^loxP/Y^* mice (8). The *TAZ* *MKO* mice were viable and fertile and displayed the expected skeletal muscle loss of *Taz* expression (Figure 3A) and elevated MLCL:CL ratio (Figure 3B) compared with littermate controls *(Taz^loxP/Y^*). Additionally, mitochondrial respiration was impaired in the *TAZ* *MKO* mice in the highly oxidative soleus muscle (Figure 3C), but mitochondrial respiration was preserved in the more glycolytic extensor digitorum longus (EDL) muscle (Figure 3D). The lack of TAZ also caused an expected decrease (36) in lean mass (Figure 3E) and a reduction in voluntary running distance (Figure 3F), which fits well with the human phenotype (1, 37). In summary, the skeletal muscle–specific TAZ-deficient mouse represents a valid in vivo model resembling the human disease for further studying our hypothesis that supplementation with omega-3 fatty acids could improve the BTHS muscle phenotype.

### Omega-3 fatty acid supplementation prevents HFD-induced loss of lean mass in TAZ MKO mice.

**Figure 4:**
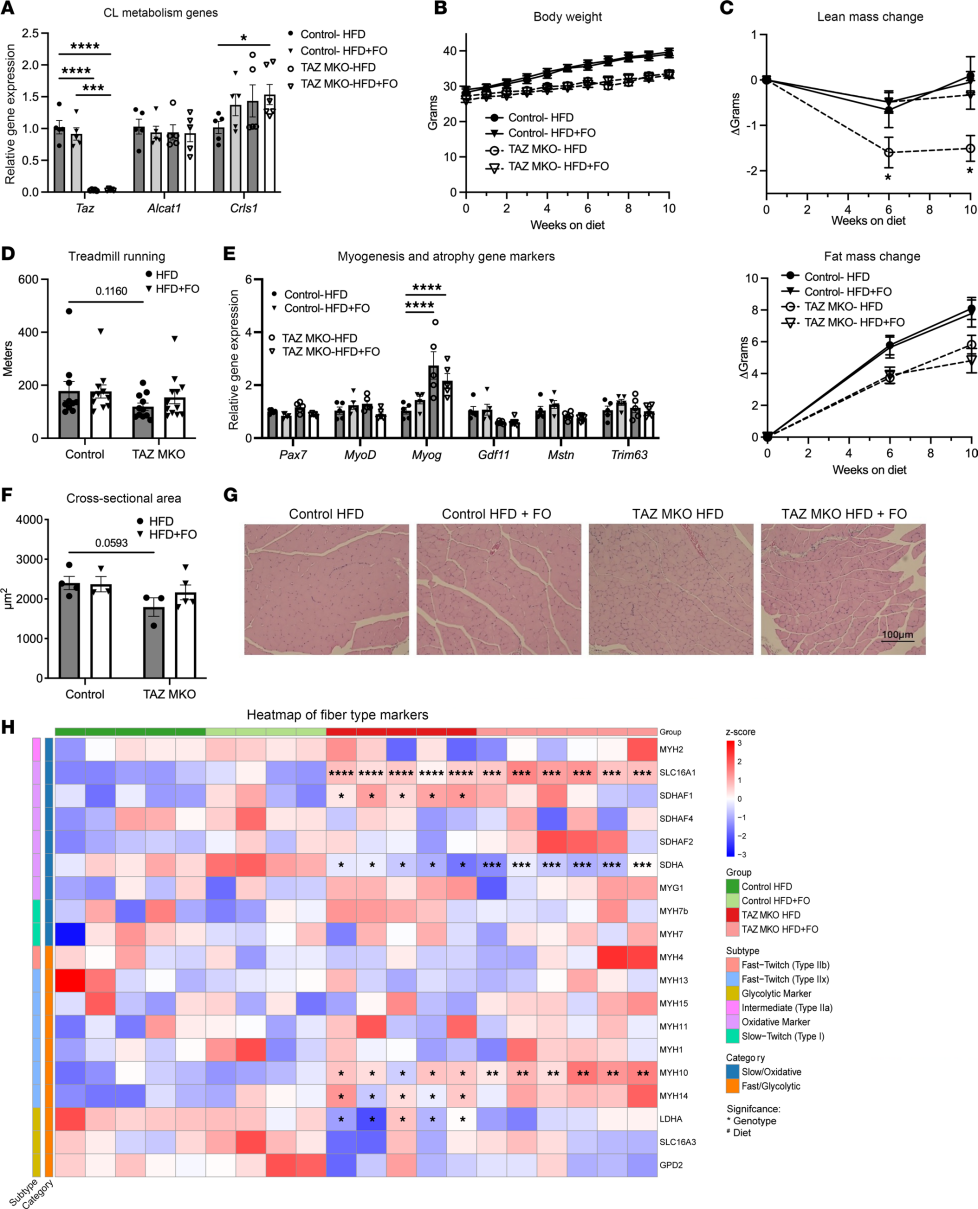
Omega-3 fatty acid supplementation prevents HFD-induced loss of lean mass in *TAZ* *MKO* mice. (**A**) Gene expression for CL metabolism–related markers in gastrocnemius (*n* = 5–6). (**B**) Body weight over 10 weeks of diet feeding (*n* = 15–21). (**C**) Change in fat and lean mass after 6 and 10 weeks of diet feeding (*n* = 15–21). (**D**) Exercise capacity on treadmills (*n* = 10–12/group). (**E**) Gene expression for myogenesis and atrophy markers in gastrocnemius (*n* = 5). (**F**) Average cross-sectional area of fibers in gastrocnemius (*n* = 3–5). (**G**) Representative H&E-stained gastrocnemius. Scale bar: 100 μm. (**H**) Heatmap of fiber type markers of proteomics analysis of gastrocnemius (*n* = 4–6). *Significant difference with the genotypte. ^#^Significant difference with the diet. Statistical significance was determined by 2-way ANOVA with the Holm-Sidak method for multiple comparison testing where appropriate. *P < 0.05; **P ≤ 0.01; ***P ≤ 0.001; ****P ≤ 0.0001.

To examine the effects of elevated dietary omega-3 fatty acids, male control and *TAZ* *MKO* littermates were placed on a high-fat Western diet (HFD) with or without 10% kcal from menhaden FO for 10 weeks. DHA and EPA accounted for approximately 6% of fatty acids or 2%–3% of kcal in the FO diet. Male mice were used because BTHS occurs nearly exclusively in males (38). An HFD was chosen to induce skeletal muscle mitochondrial impairment and stress, and to ensure mice would eat the FO-containing diet. The diet did not affect *Taz* or *Alcat1* expression, and *Crls1* was elevated in *TAZ* *MKO* mice fed FO (Figure 4A). Over the 10-week feeding study, *TAZ* *MKO* mice gained less body weight and fat mass regardless of diet (Figure 4, B and C). Despite having lower baseline lean mass (Figure 3E), *TAZ* *MKO* mice fed the normal HFD lost more lean mass over the 10-week feeding period (Figure 4C). Importantly, *TAZ* *MKO* mice supplemented with FO maintained lean mass, similar to control mice (Figure 4C). Surprisingly, no substantial impairment in the endurance exercise capacity for forced treadmill running was observed with loss of TAZ in skeletal muscle (Figure 4D), indicating that the observed major defect in exercise capacity in the whole-body TAZ KO (11) may be due to defects in heart function. Additionally, excess adiposity may hamper exercise capacity in the control mice fed the HFD (39), and counter potential effects of lacking TAZ in skeletal muscle per se on exercise capacity. Expression of myogenesis- and atrophy-related genes revealed no interaction between diet and genotype, but *Myog* expression was elevated and *Gdf11* was reduced in *TAZ* *MKO* gastrocnemius regardless of diet (Figure 4E). The gastrocnemius of *TAZ* *MKO* HFD-fed mice displayed slightly smaller cross-sectional area of the myocytes, which was normalized with FO feeding (Figure 4, F and G). Protein analysis of different fiber types revealed no changes in the subtype-determining markers with the FO diet or genotype, and no pattern towards a drastic change in a specific fiber subtype enrichment (Figure 4H). Together, these data indicate that omega-3 fatty acid supplementation prevents lean mass loss and normalized muscle fiber myocyte size, several of the impaired phenotypes central to BTHS (37, 38, 40).

### Impaired mitochondrial respiration in TAZ MKO soleus can be rescued by omega-3 fatty acid supplementation.

**Figure 5:**
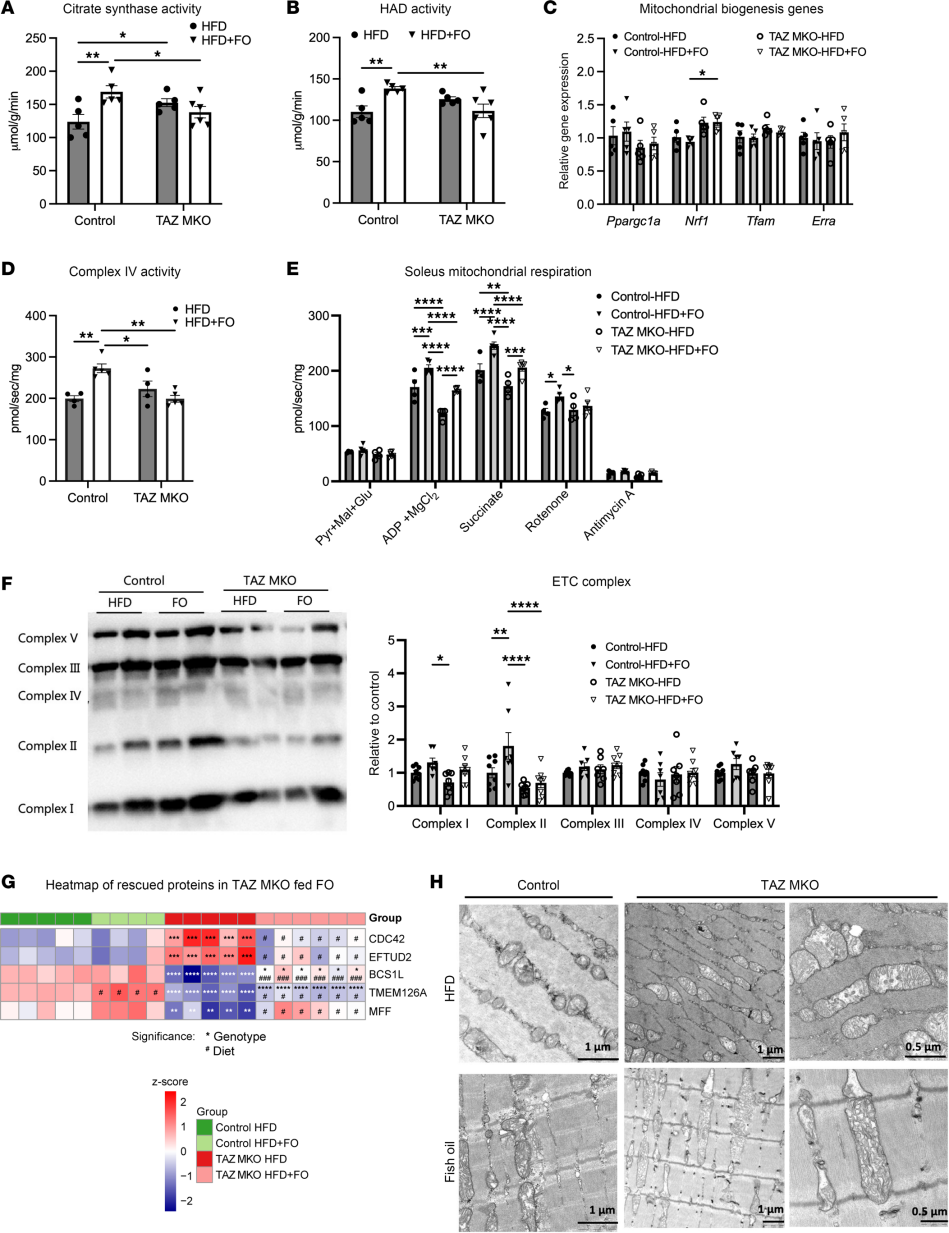
Impaired mitochondrial respiration in *TAZ* *MKO* soleus can be rescued by omega-3 fatty acid supplementation. (**A** and **B**) Citrate synthase and β-hydroxy acyl-CoA dehydrogenase (HAD) activity in soleus after 10 weeks of diet feeding (*n* = 5–6). (**C**) Expression of genes related to mitochondrial biogenesis in gastrocnemius (*n* = 5). (**D** and **E**) High-resolution respirometry in soleus after 10 weeks of diet feeding (*n* = 4–5). (**F**) Representative image of mitochondrial oxidative phosphorylation complexes and densitometry (*n* = 7–8). (**G**) Heatmap of rescued proteins of the gastrocnemius in the *TAZ* *MKO* proteome fed fish oil. *Significant difference with the genotype. ^#^Significant difference with the diet. (**H**) Electron microscopy to show mitochondrial structure in soleus. Scale bars: 1 μm (4 left images) and 0.5 μm (2 right images) (*n* = 1). Statistical significance was determined by 2-way ANOVA with the Holm-Šídák method for multiple comparison testing where appropriate. *P < 0.05; **P ≤ 0.01; ***P ≤ 0.001; ****P ≤ 0.0001.

Soleus was chosen for further analysis due to its high mitochondrial content and dependence on oxidative metabolism, as well as its apparent mitochondrial respiratory defects when lacking TAZ (Figure 3C). FO feeding of control mice increased mitochondrial content and fatty acid oxidative capacity, as indicated by citrate synthase (CS) and β-hydroxy acyl-CoA dehydrogenase (HAD) activity, respectively (Figure 5, A and B). Interestingly, HFD-fed *TAZ* *MKO* mice had higher mitochondrial content than control mice (Figure 5A), suggesting potential compensation for impaired mitochondrial function. This parameter was normalized with FO feeding in the *TAZ* *MKO* mice, which importantly means that potential changes to respiration are not due to FO-fed *TAZ* *MKO* mice having more dysfunctional mitochondria. Expression of transcription factors related to mitochondrial biogenesis was not affected by diet, but a general elevation in *TAZ* *MKO* mice was observed for *Nrf1* (Figure 5C), indicating that a reduction in mitochondrial content in FO-fed *TAZ* *MKO* soleus was not due to impaired mitochondrial biogenesis signaling. No differences were seen in complex IV activity in *TAZ* *MKO* mice (Figure 5D), but HFD-fed *TAZ* *MKO* mice had impaired ADP-stimulated respiration (Figure 5E). Importantly, when fed FO to increase dietary omega-3 fatty acids, ADP- and succinate-stimulated respiration were normalized in *TAZ* *MKO* mice to levels of HFD-fed control mice (Figure 5E), indicating a rescue of mitochondrial respiratory capacity. At an earlier time point, after 5 weeks of FO feeding, we already observed a slight improvement in *TAZ* *MKO* ADP- and succinate-stimulated respiration without affecting respiration in the control mice (Supplemental Figure 3).

CL is important for the structure of the inner mitochondrial membrane and protein incorporation, especially for the electron transport chain (41–43). Protein levels of mitochondrial complex I and II were lower in *TAZ* *MKO* HFD-fed mice compared with controls, and FO feeding normalized complex I levels in the *TAZ* *MKO* soleus (Figure 5F), indicating one potential mechanism for the improved respiration with FO feeding in *TAZ* *MKO* mice. A proteome assessment of the gastrocnemius revealed that the genotype drives changes in the protein distribution (Supplemental Figure 4, A–D). However, several proteins in the gastrocnemius of *TAZ* *MKO* mice fed FO were normalized to the control condition. These proteins included 3 mitochondria-associated proteins: MFF, TMEM126A, and BCS1L (Figure 5G), supporting the observed functional rescue.

When comparing structures of interfibrillar mitochondria, we observed highly disrupted cristae with more open areas in *TAZ* *MKO* mice fed HFD, and unusual cristae patterns in *TAZ* *MKO* mice fed HFD plus FO (Figure 5H). These images indicate that, while FO feeding improved function, it did not fully normalize the mitochondrial structure, suggesting an abnormal compensation of membrane lipids. In summary, dietary FO supplementation appears to restore mitochondrial respiratory capacity in *TAZ* *MKO* mice without increasing mitochondrial number.

### TAZ deficiency affects cellular health and FO modulates only some markers of stress and damage.

**Figure 6:**
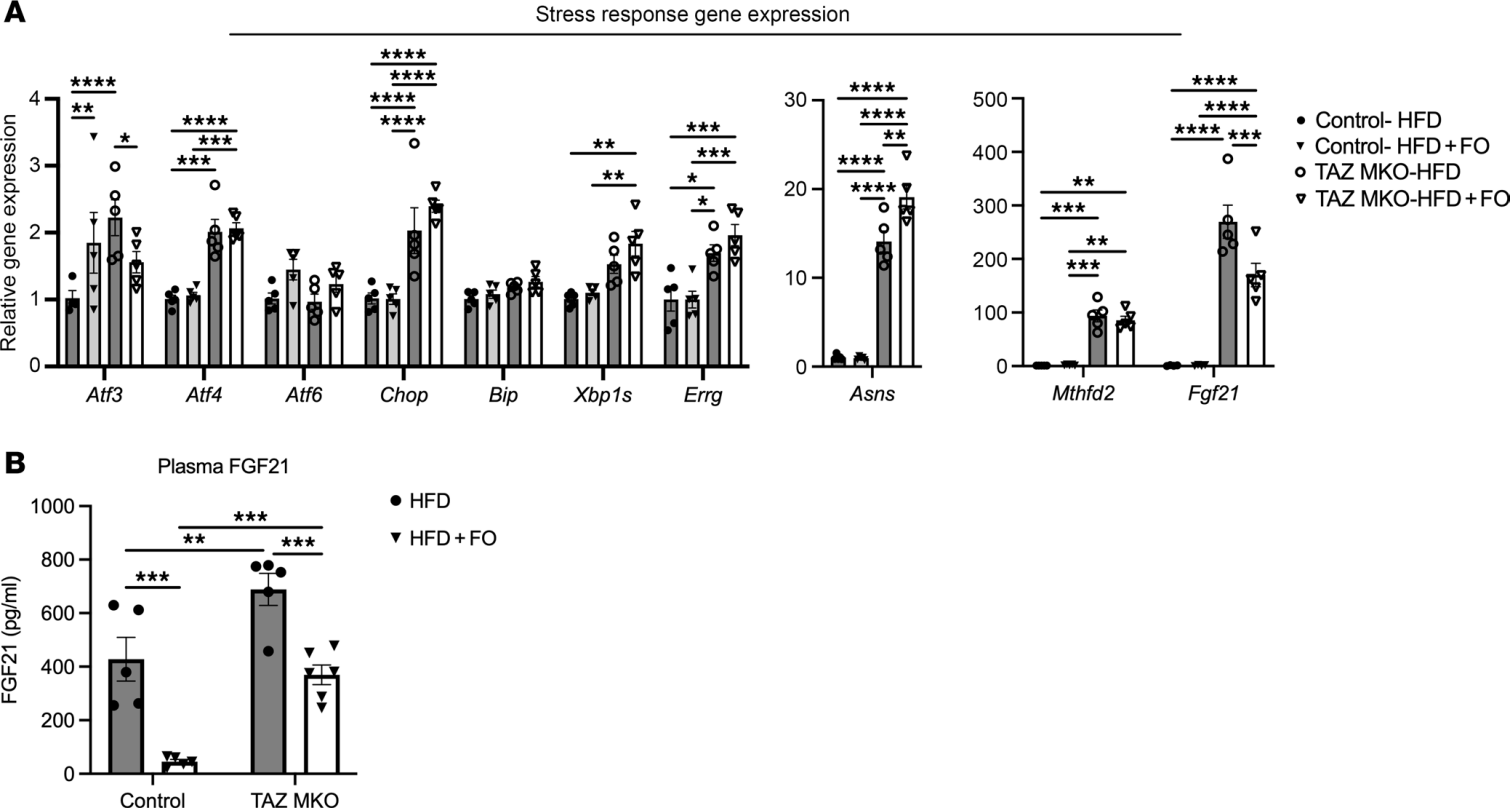
TAZ deficiency affects cellular health and fish oil modulates only some markers of stress and damage. (**A**) Gene expression of stress-response markers in gastrocnemius (*n* = 5). (**B**) Plasma FGF21 (*n* = 5–6). Statistical significance was determined by 2-way ANOVA with the Holm-Šídák method for multiple comparison testing where appropriate. *P < 0.05; **P ≤ 0.01; ***P ≤ 0.001; ****P ≤ 0.0001.

In accordance with impaired muscle mitochondrial function in *TAZ* *MKO* mice fed HFD, the stress response seemed highly activated in the gastrocnemius muscle, a mixed-fiber type muscle (Figure 6A), with some indication that FO feeding improved this response indicated by the observed 37% reduction in *Fgf21* gene expression. BTHS patients have elevated plasma FGF21 protein (44), and in line with this, the *TAZ* *MKO* HFD-fed mice also displayed elevated plasma FGF21 levels, which was normalized with FO feeding (Figure 6B). Because the liver retains functional TAZ in this model and the muscle expression of *Fgf21* is highly elevated in *TAZ* *MKO* mice, it is likely that the elevation in circulating FGF21 levels is due to secretion from mitochondrially compromised muscle cells, as evident in muscle-specific mouse models with mitochondrial deficiencies (45–47). Interestingly, FO feeding lowered plasma FGF21 in both genotypes, indicating a reduced systemic cellular stress (48) in addition to those effects occurring in the skeletal muscle.

### Loss of TAZ in skeletal muscle prevented diet-induced changes in CL species.

**Figure 7:**
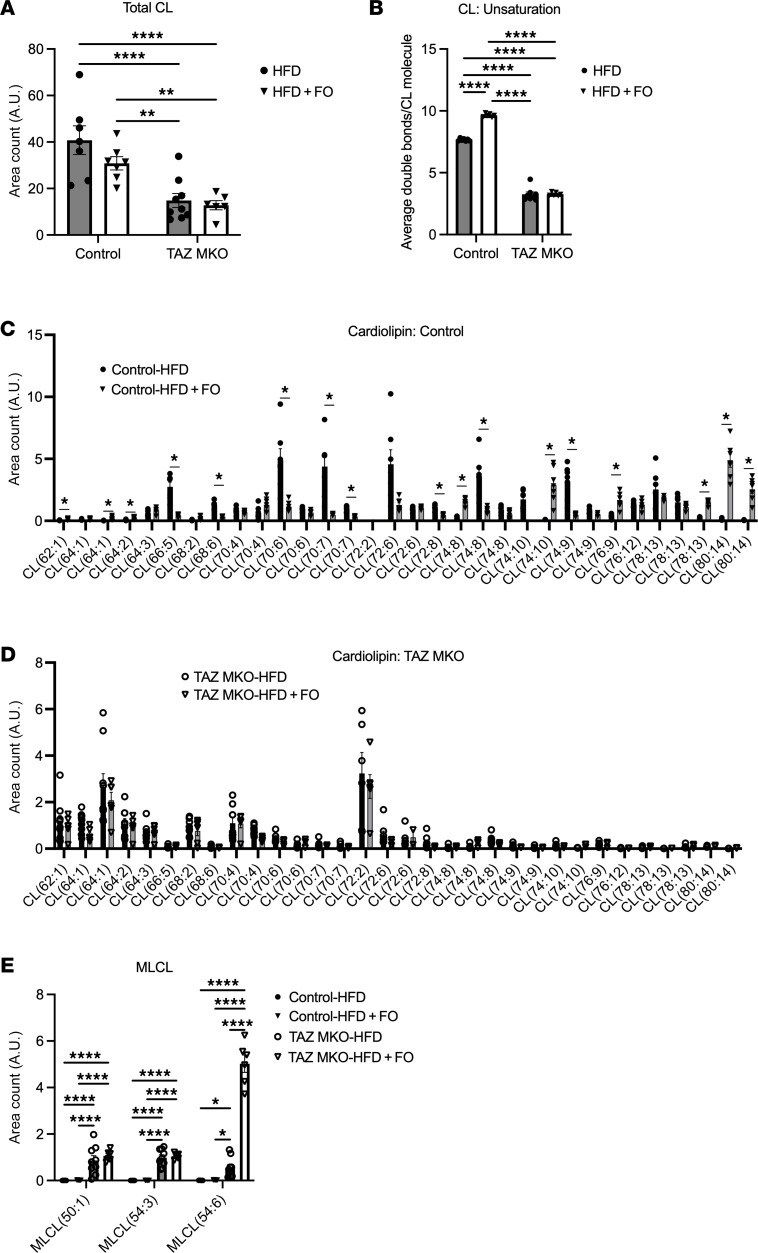
Loss of TAZ in skeletal muscle prevented diet-induced changes in cardiolipin species. (**A**) Total area assigned to cardiolipin (CL) in soleus (*n* = 6–9). (**B**) Unsaturation of acyl chains relative to area of CL (*n* = 6–9). (**C** and **D**) CL species in control (**C**) and *TAZ* *MKO* (**D**) soleus after 10 weeks of diet feeding (*n* = 6–9). (**E**) Monolyso-CL (MLCL) species (*n* = 6–9). Statistical significance was determined by 2-way ANOVA (**A**, **B**, and **E**) or multiple unpaired *t* tests (**C** and **D**) with the Holm-Šídák method for multiple comparison testing where appropriate. *P < 0.05; **P ≤ 0.01; ****P ≤ 0.0001.

As we observed abnormal muscle mitochondrial structure despite improved mitochondrial function upon FO feeding in *TAZ* *MKO* mice (Figure 5G), we hypothesized that structural lipids might be altered. Therefore, we analyzed the whole-cell lipids of the soleus by LC-MS and observed that *TAZ* *MKO* muscle had low CL content, regardless of diet (Figure 7A). FO feeding of control mice shifted the composition of CL species towards longer, more unsaturated species (Figure 7, B and C). Interestingly, FO feeding did not change the CL composition of the *TAZ* *MKO* mice (Figure 7, B and D), indicating that even the nascent CL was likely unaffected. Additionally, MLCL was elevated in *TAZ* *MKO* mice, regardless of diet (Figure 7E). Therefore, the observed improvements in lean mass and mitochondrial respiration in *TAZ* *MKO* mice with FO feeding were not due to normalization of CL or reduction in MLCL in the soleus.

### TAZ deficiency alters many phospholipids, with differing responses to FO.

**Figure 8:**
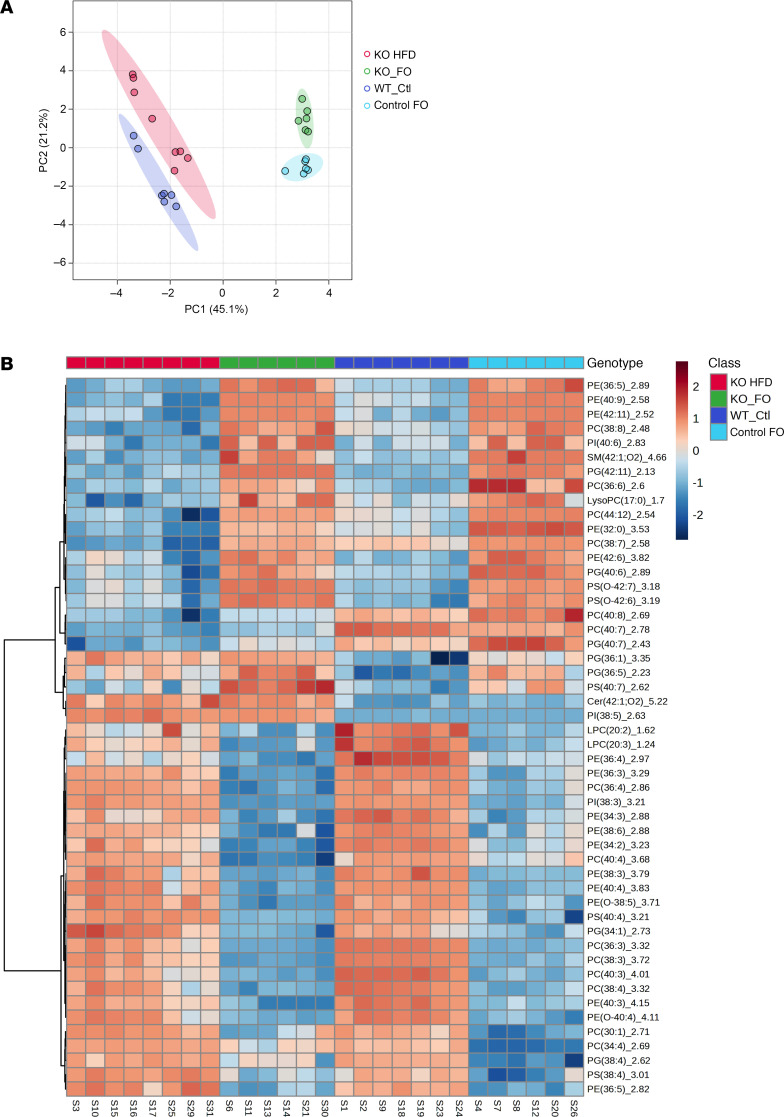
TAZ deficiency affects many phospholipids with differing responses to fish oil. (**A**) Principal component analysis (PCA) plot after removing cardiolipin (CL) and monolyso-CL (MLCL) species from the lipidomics data (*n* = 6–9). (**B**) Heatmap of the top 50 changes in phospholipid species (*n* = 6–9). Analyzed with MetaboAnalyst.

We further investigated the other phospholipids to better understand the effects on membrane lipids. After removal of CL and MLCL from the analysis, the lipidome of the 4 different groups remained distinct (Figure 8A), emphasizing the broader impact of TAZ deficiency beyond its typical effects on CL, in line with our hypothesis that additional membrane lipids are altered. Both diet and lack of TAZ in skeletal muscle altered the lipidome, and while we cannot determine causality in the current model, several differences were of note. Most phospholipids were changed only at the species composition, but total phosphatidylserine (PS) was higher in *TAZ* *MKO* mice and was normalized to control levels with FO supplementation (Supplemental Figure 5A). The degree of unsaturation of the different phospholipid classes was largely affected by diet, with the CL species in *TAZ* *MKO* mice being a notable exception (Figure 8B and Supplemental Figure 5B). Several ceramides had a strong genotype effect, with partial normalization by FO feeding in the *TAZ* *MKO* mice (Supplemental Figure 5C). Consistent with this, the abundance of the hexosylceramide species HexCer (44:0; O2) was elevated in *TAZ* *MKO* mice but normalized to a similar level as seen in control groups with FO feeding of *TAZ* *MKO* mice (Supplemental Figure 5D). Additionally, PI (38:5) was detected at low levels in control mice, but was abundant in all *TAZ* *MKO* muscle. Interestingly, this PI was reduced by FO feeding in the *TAZ* *MKO* mice (Figure 8B and Supplemental Figure 5D). These results provide clues to the mechanisms behind the rescue of mitochondrial respiration in *TAZ* *MKO* mice as well as a resource to understand both the effects of loss of TAZ and omega-3 fatty acid supplementation in skeletal muscle.

## Discussion

BTHS is a rare disease affecting the skeletal muscle, heart, and immune system in young children (1, 49), with limited treatment options and impaired quality of life (12). Here, we investigate a model of a constitutive loss of TAZ in skeletal muscle. This model has both advantages and disadvantages compared with other models of BTHS; however, it allows assessing the contribution of skeletal muscle TAZ to the phenotype observed in BTHS. The earliest murine model of BTHS required doxycycline treatment to knock down TAZ (50, 51), but doxycycline-induced knockdown has several disadvantages, including impairment of mitochondrial function (52) and extracellular matrix (53), but much was learned through the whole-body knockdown. The constitutive, whole-body knockout of TAZ has high early lethality (8, 54), making it difficult to study in the surviving mice. A recent study of BTHS patients revealed that heart transplantation did not improve any skeletal muscle–related disease characteristics, such as enhanced exercise tolerance or muscle mass (30), suggesting that skeletal muscle has an independent effect on the pathology. Thus, the model generated and used here, *TAZ* *MKO*, is highly relevant to study skeletal muscle–specific effects in BTHS. Our model recapitulates the lack of functional TAZ and loss of CL in skeletal muscle seen in patients with BTHS, as well as impaired mitochondrial respiration, reduced lean mass (37, 38, 40), and elevated circulating FGF21 levels (44). Therefore, *TAZ* *MKO* represents a suitable model to study the effects of the lack of TAZ specifically in skeletal muscle.

In this study, we show that a simple dietary change led to important improvements in the skeletal muscle lacking the gene responsible for BTHS. By incorporating more omega-3 fatty acids into the diet in the form of FO, *TAZ* *MKO* mice displayed improved mitochondrial respiration and prevention of lean mass loss, major factors in the development of BTHS. Elevation of omega-3 fatty acids with the diet has been shown to have beneficial effects on cellular and metabolic stress (14, 55, 56). FGF21 reduction in *TAZ* *MKO* mice fed the FO-containing diet may be even more pronounced and potentially cardioprotective, in a cardiac-specific or whole-body TAZ KO with the dietary intervention. Neonates can tolerate supplementation with omega-3 fatty acids (57), highlighting feasibility to start early dietary supplementation with omega-3 fatty acids. If future work can confirm the benefit of omega-3 fatty acids to hearts lacking TAZ, it would be ideal to test omega-3 fatty acids as an addition to treatment of BTHS, as the supplements are low cost, easy to obtain, have few side effects, and can be given in child-friendly formats.

Additionally, this study highlights the importance of other phospholipids beyond CL in mitochondrial function in BTHS. Surprisingly, the improvement in mitochondrial respiration in FO-fed *TAZ* *MKO* mice was not due to an increase in CL or decrease in MLCL. This finding indicates that other phospholipids likely compensate for the lack of CL when sufficient omega-3 fatty acids are present. Changes in phospholipids with a different shape than CL may be the cause of the abnormal cristae shape (Figure 5H), but these changes appear sufficient to rescue complex I protein and improve respiration. For instance, a higher degree of unsaturation in PEs could potentially impact membrane fluidity and support respiratory complex activity (58). Additionally, normalization of other lipids, such as hexosylceramides, which have been associated with impaired mitochondrial function and cellular stress (59), may contribute to the improved phenotype. Many phospholipids were altered, but this study was not designed to tease out whether a class or other group of phospholipids is responsible for the partial improvement in overall structure and function. Future work is needed to better understand this compensation, as it may also be relevant within aging, which is associated with reduced CL (60).

This study also highlights the cell-specific nature of TAZ deficiency. In HEK293 and C2C12 cells, supplemental omega-3 fatty acids were able to be incorporated into CL to compensate for the lack of LA, but this finding was not observed in the skeletal muscle of the *TAZ* *MKO* mice. Similarly, in fibroblast cells of BTHS patients, CL remodeling was possible by the addition of a high dose of LA (61), which we could not observe in HEK cells lacking TAZ with a moderate dose of LA. Moreover, the in vivo confirmation of LA incorporation in CL when TAZ-deficient mice were given dietary LA is controversial (23–25). Nevertheless, functional improvement of the phenotype in BTHS could not be recapitulated with dietary LA supplementation (25). In a complex system, there are both tissue interactions and tissue-specific reasons to consider, and the regulatory complexity of an intact organism differs fundamentally from that of a simplified in vitro system. Delivery of omega-3 fatty acids from chylomicrons or liver-derived very low-density lipoprotein is different from an albumin-bound free fatty acid in cell culture. Additionally, skeletal muscle shows low expression of another CL remodeling enzyme (ALCAT) (62) that could compensate in other tissue types and in the cell lines used. This might help explain the tissue-specific nature of BTHS, as other tissues are less affected than heart and skeletal muscle, which may be due to compensation and use of other fatty acids.

Increasing dietary omega-3 fatty acids offers positive effects on cellular stress as well as the immune system (14, 55, 56), both of which are crucial for overall health and may be specifically important to patients with BTHS. In particular, omega-3 fatty acids affect neutrophil function and act as antioxidants (63). Increased oxidative stress has been observed in different BTHS models (32, 35, 64) and omega-3 fatty acid supplementation has been shown to improve oxidative stress parameters (55). Neutropenia in BTHS occurs in more than 80% of patients (2, 65, 66) and is associated with bacterial infections, one of the leading causes of death (2). Thus, omega-3 fatty acids could support antiinflammatory responses during infection (67) and neutrophil function, as FO supplementation even prevented chemotherapy-induced decline in neutrophil number and function (68). The positive effects of omega-3 fatty acids or FO supplementation could improve various aspects of the BTHS pathology, potentially yielding improvements in life quality. These aspects of omega-3 fatty acid supplementation in BTHS should be investigated in future studies.

In conclusion, we generated a mouse model to study the loss of TAZ specifically in skeletal muscle replicating the phenotype of BTHS with decreased lean mass and mitochondrial function, reduced voluntary exercise, as well as increased muscular and cellular stress markers. By elevating dietary omega-3 fatty acids, we prevented lean mass loss and improved mitochondrial function in *TAZ* *MKO* mice by altering mitochondrial structure and muscular phospholipid composition without modification of MLCL or CL composition. Here, we provide a potential avenue, to improve the phenotype, quality of life, and outcome in people with BTHS that could be added to current treatment regimes.

## Methods

### Sex as a biological variable.

Our study exclusively examined male mice because the disease modeled (BTHS) is primarily relevant in males (2, 38, 69), as female carriers are mostly asymptomatic (49, 70). Investigated improvements are potentially relevant for the target group of males suffering from BTHS, but may not be irrelevant for affected females.

### Cell culture.

HEK293 Flp and T*az*^TALEN^.19 (*TAZ* *KO*) (31) cells were cultured to confluence in 25 mM glucose DMEM with 10% FBS. Cells were incubated with a relatively physiologic mixture of 25 μM oleate,15 μM palmitate, 5 μM linoleate, 5 μM arachidonate or 5 μM docosahexaenoic acid bound to bovine serum albumin (BSA). For labeling, 0.5 μCi of [1-^14^C]oleate, [1-^14^C]linoleate, [1-^14^C]arachidonate, or [1-^14^C]docosahexaenate was substituted for the appropriate amount of unlabeled fatty acid. Incubations lasted for 24 hours and then cells were washed twice with 0.1% BSA. For pulse-chase experiments, cells were incubated with the fatty acid mix and labeled fatty acid media for 4 hours (pulse), washed once with 0.1% BSA, and incubated with media containing an unlabeled fatty acid mixture for 20 hours. For oxidation measurements, 0.4 mL media was collected in a tube containing 20 μL 15% BSA and then incubated with 100 μL 20% perchloric acid overnight at 4°C. The acidified media was centrifuged at 20,000*g* for 5 minutes, and radioactivity in the supernatant was counted to determine acid-soluble metabolites. Lipids were extracted from cells using chloroform and methanol (71), and phospholipids were separated by thin layer chromatography (TLC) in chloroform/ethanol/water/triethylamine (30:35:7:35; v/v) with authentic standards (72). Radioactivity was quantified using a TLC scanner (AR-2000, Eckert-Ziegler).

*TAZKO* and WT control C2C12 cells (35) were a gift from Miriam Greenberg (Wayne State University, Detroit, Michigan, USA), and were maintained in high-glucose (25 mM) DMEM supplemented with 10% FBS and 1% penicillin/streptomycin. Differentiation of myoblasts was induced by switching the media to high-glucose DMEM without sodium pyruvate, supplemented with 2% horse serum and 1% penicillin/streptomycin. Media were refreshed every second day during the 7-day differentiation period. Differentiated cells were treated with either 50 μM BSA-conjugated DHA+EPA–containing fatty acid mix (7.5 μM DHA, 13.4 μM EPA, 29.1 μM oleate according to Banic et al. (73), with a ratio of EPA to DHA of 1.8:1 as in menhaden FO) or 50 μM BSA-conjugated oleate from day 7 to day 9, and the medium was replaced every day. Cells were washed with PBS containing 1% fatty acid–free BSA, harvested in 140 μL of 150 mM NH_4_OAc, snap-frozen in dry ice, and stored at –80°C until analysis.

### Mouse model.

All mouse protocols were approved by the Danish Animal Experiments Inspectorate and performed according to Animal Research: Reporting of In Vivo Experiments (ARRIVE) standards. Mice were housed in a specific pathogen–free environment on a 12-hour light/12-hour dark cycle and were maintained on a chow diet with ad libitum access to food and water unless otherwise specified. Skeletal muscle–specific *TAZ* *MKO* mice were generated by crossing the *Taz^loxP^* strain (8) (provided by Douglas Strathdee, Cancer Research UK, London, United Kingdom) with a constitutively *HSA-cre*–expressing line (74). All mice were fully floxed and on a C57BL/6Ntac background. *TAZ* *MKO* mice were viable and fertile. Male mice were used for experiments, unless otherwise specified, as BTHS primarily occurs in male humans (2, 38, 69). At 8–12 weeks of age, male mice were placed on either an HFD containing 10% menhaden FO (4.7 kcal/g; 40% kcal fat, 17% kcal protein, 43% kcal carbohydrate, D22060303, Research Diets) or matched control diet (D22060302, Research Diets). DHA and EPA content in the HFD+FO diet was approximately 6% of total fatty acids, comparable to the approximately 3% of added fatty acids in the cell culture experiments. Following a 1-week acclimation period to individual housing with a running wheel, voluntary running was measured daily for 1 week and expressed as the average of the 7 days. Body composition was measured by MRI (Bruker LF90II MRI scanner) at baseline, 6 weeks, and 10 weeks of the diet study. Tissues and blood were collected from pentobarbital-anesthetized mice after 10 weeks and either snap frozen or processed further.

### TBARS assay.

Malondialdehyde content was determined in cell lysates and measured as thiobarbituric acid–reactive substance (TBARS) according to the manufacturer’s recommendation (10009055, Cayman Chemical).

### Exercise capacity testing.

Exercise capacity was measured as previously described (75) after 10 weeks on respective diets. Briefly, mice were acclimatized to the treadmill and running for 3 days and then rested for 1 day. Exercise capacity testing was conducted with 5% incline, starting at 6 m/min with increases in speed every 2 minutes until max speed of 16 m/min, with a maximum running time of 60 minutes. Exhaustion was defined as refusal to run for 5 seconds while in contact with the shock grid or after 3 shocks.

### Histology.

Gastrocnemius muscle was fixed in 4% paraformaldehyde and then embedded in paraffin, cut cross-sectionally, and stained with hematoxylin and eosin (H&E). The slides were visualized with an AXIO Lab A1 Light Microscope (Zeiss), bright-field, 20× objective magnification. Ten images of each sample were taken with AxioCam ICc 5 (Zeiss). The cross-sectional area of cells was measured using FIJI (ImageJ).

### Mitochondrial function measurements.

Mitochondrial respiratory capacity was measured in permeabilized skeletal muscle fibers of soleus (Figure 3C, Figure 5E, and Supplemental Figure 3) and EDL (Figure 3D) using high-resolution respirometry (Oxygraph-2k, Oroboros). Skeletal muscle tissue was placed in BIOPS buffer (76) after dissection and kept on ice. The skeletal muscle tissue was mechanically dissected on ice using sharp forceps, chemically permeabilized in BIOPS buffer containing saponin (50 μg/mL) for 30 minutes, and then washed twice for 10 minutes in MiR05 buffer. Skeletal muscle fibers were weighed and added to the respirometry chamber containing MiR05 buffer.

The following protocol was performed in duplicate at 37°C after hyperoxygenation (450–200 nmol/mL) to avoid potential oxygen limitation. Pyruvate (5 mM), malate (2 mM), and glutamate (10 mM) (LEAK) were added to each chamber, followed by ADP (5 mM) and MgCl_2_ (3 mM) to determine maximal complex I respiration (CI*_P_*). Cytochrome *c* (0.01 mM) was added to verify the integrity of the outer mitochondrial membrane. Succinate (10 mM) was applied to assess the maximal respiration in both complex I and II (CI+II*_P_*). Subsequently, titration of rotenone (0.001 mM) and antimycin A (0.005 mM) was performed to determine the residual oxygen consumption respiration. The chambers were reoxygenated before addition of ascorbate (2 mM) and TMPD (0.5 mM) to assess cytochrome *c* oxidase (CIV) activity. The oxygen concentration at the time point, after the respiratory flux rate started to decline, was noted. Azid (100 mM) was applied, and the oxygen concentration was raised to the same level as noted in the previous step to correct for the autoxidation of TMPD and ascorbate under the given oxygen pressure.

CS and HAD activity was measured as previously described (77) with some minor changes. Five milligrams of wet weight skeletal muscle was homogenized in 800 μL homogenization buffer with 8 μL 10% Triton X-100. CS activity was used to estimate mitochondrial number.

### Electron microscopy.

Mice were perfused through the left ventricle with 2% v/v glutaraldehyde in 0.05 M sodium phosphate buffer (pH 7.2) and stored in this fixation mixture. Following isolation of suitable specimen blocks, the samples were rinsed 3 times in 0.15 M sodium phosphate buffer (pH 7.2) and subsequently postfixed in 1% w/v OsO_4_ with 0.05 M K_3_Fe(CN)_6_ in 0.12 M sodium phosphate buffer (pH 7.2) for 2 hours. The specimens were dehydrated in graded series of ethanol, transferred to propylene oxide, and embedded in Epon according to standard procedures. Sections, approximately 60 nm thick, were cut with an Ultracut 7 (Leica) and collected on 1-hole copper grids with Formvar supporting membranes, stained with uranyl acetate and lead citrate, and subsequently examined with a Philips CM 100 transmission electron microscope, operated at an accelerating voltage of 80 kV. Digital images were recorded with an OSIS Veleta digital slow scan 2k × 2k CCD camera and the ITEM software package.

### Western blot analysis.

Soleus was homogenized in ice-cold homogenization buffer (10% glycerol, 1% NP-40, 20 mM sodium pyrophosphate, 150 mM NaCl, 50 mM HEPES [pH 7.5], 20 mM β-glycerophosphate, 10 mM NaF, 2 mM phenylmethylsulfonyl fluoride [PMSF], 1 mM EDTA [pH 8.0], 1 mM EGTA [pH 8.0], 2 mM Na_3_VO_4_, 10 μg/mL leupeptin, 10 μg/mL aprotinin, 3 mM benzamidine) using a Qiagen Tissue-Lyser II (60 seconds at 30 Hz), after which the homogenate was placed end-over-end for 30 minutes at 5°C. The samples were then centrifuged at 10,000*g* for 15 minutes at 4°C and the supernatant (lysate) was collected discarding the remaining pellet and frozen at –80°C until further analyses. Lysate protein concentration was determined using the bicinchoninic acid (BCA) method. Soleus lysate (8 μg protein/lane) was separated using SDS-PAGE on a 4%–15% gradient gel and transferred to a PVDF membrane under standard conditions. The membrane was blocked in 3% BSA in TBST and then incubated with a primary antibody against OXPHOS complexes (Abcam, ab110413; 1:5,000) in TBST with 3% BSA, overnight at 4°C. After washing, the membrane was incubated with secondary antibody rabbit anti-mouse–HRP (Jackson ImmunoResearch, 115-035-062; 1:5,000 in TBST with 3% BSA) for 1 hour at room temperature. Bands were visualized using chemiluminescence using a Bio-Rad ChemiDoc MP Imaging System and enhanced chemiluminescence (ECL). Densitometry was analyzed with Image Lab software.

### Proteomics.

Approximately 10 μg of gastrocnemius muscle was lysed in 50 mM HEPES pH 8.5 containing 5% sodium deoxycholate, supplemented with cOmplete protease inhibitor (EDTA-free 118735800001, Roche). Samples were sonicated for 30 seconds on ice, and afterward, proteins were denatured at 98°C for 5 minutes. Samples were then centrifuged at 20,000*g* for 20 minutes at 4°C, and the supernatant was transferred to a new tube. Protein concentrations were determined by BCA. Samples were reduced with 10 mM dithiothreitol and alkylated with 20 mM iodoacetamide for 20 minutes each. An aliquot of 20 μg of protein was digested at 37°C after preincubation with LysC (0.04 AU/mg protein), then 5% trypsin was added overnight. Sodium deoxycholate was precipitated by adding TFA to a final concentration of 1% and then centrifuged at 20,000*g* for 10 minutes. Afterward, the supernatant was collected, peptides were purified using C18 stage tips, and the samples were then vacuum-dried. Finally, peptides were resuspended in 0.1% formic acid.

Approximately 1,000 ng of peptides, dissolved in solvent A (0.1% formic acid), was loaded onto a 20-cm analytical column (100-μm inner diameter) packed with ReproSil-Pur C18 AQ 1.9-μm RP material using a Thermo Scientific Vanquish Neo UHPLC system coupled to an Orbitrap Astral mass spectrometer. Elution of peptides was performed at a flow rate of 300 μL/min by increasing the percentage of solvent B (95% acetonitrile and 0.1% formic acid) from 0.3% to 38% over 40 minutes, then to 90% over 2 minutes, and holding at 90% for 5 more minutes. During the 30-minute gradient, the mass spectrometer operated in positive mode with Orbitrap resolution set at 240,000, a scan range of 400–1000 *m*/*z*, and a maximum injection time of 5 ms. Data-independent acquisition (DIA) was performed with higher-energy collision-induced dissociation set to 25%, a scan range of 150–2000 *m*/*z*, a maximum injection time of 3.5 ms, and a cycle time of 0.6 seconds. Fragment ions were detected using the Astral detector.

Raw data files were searched with Spectronaut 20.0 (https://biognosys.com/software/spectronaut/), using the *Mus* *musculus* reference proteome FASTA file, ID UP000000589, containing 21,757 genes (downloaded on 27.01.2025) (https://www.uniprot.org/proteomes/UP000000589). BGS factory settings were used for the search, with minor modifications. Most search settings were kept at their defaults. In brief, search settings included trypsin/P-digested peptides with a maximum of 2 missed cleavages, carbamidomethyl (C) as a fixed modification, and a maximum of 5 variable modifications, including acetyl (protein N-term) and oxidation (M). The Deep directDIA^+^ workflow was used for identification. For quantification, precursor filtering was set to “Identified (Qvalue),” and no imputation was performed. The proteotypicity filter was set to “Only protein group specific,” and cross-run normalization was used.

Bioinformatic analyses were performed in R (4.5.0) (https://cran.r-project.org) within a reproducible environment managed by the renv package. Protein intensities obtained after Spectronaut search were imported with readr. The dataset was filtered to retain only proteins with at least 3 valid values in each experimental group (WT_HFD, WT_FO, KO_HFD, KO_FO), and intensities were log_2_-transformed by DEP:make_se(). Missing values were imputed using the MinProb method (quantile *q* = 0.01) from the DEP package with a fixed random seed [123] set before imputation to ensure reproducibility. Differential protein abundance was determined using the limma package and a 2 × 2 factorial experimental design (genotype: WT/KO; diet: HFD/FO). Five contrasts were defined to test the genotype effect within each diet (KO − WT in HFD; KO − WT in FO), the diet effect within each genotype (HFD − FO in WT; HFD − FO in KO), and the genotype × diet interaction. Empirical Bayes moderation was applied to the model to increase statistical power, and *P* values were adjusted for multiple testing using the Benjamini-Hochberg false discovery rate (FDR) procedure. Proteins with an FDR of less than 5% and log_2_(fold change) of greater than 0.5 were considered statistically significant. Volcano plots were generated with ggplot2, and heatmaps were generated with pheatmap.

### FGF21 measurement.

Blood was centrifuged 8,000*g* for 10 minutes at 4°C to separate plasma. FGF21 was measured in plasma using the Mouse FGF-21 ELISA (Abcam, ab212160), according to the manufacturer’s instructions.

### Lipidomics.

Lipidomics analyses were performed essentially as described previously (78). Briefly, cell or tissue lipids were extracted using Folch extraction (71). Prior to tissue lysis, SPLASH mix (Merck) was added to the extraction solvent, and tissue samples were lysed by beat beating in a FastPrep-24 homogenizer. After centrifugation and phase separation, the apolar and polar phases were transferred to separate tubes, and the apolar phase dried under N_2_. Samples were resuspended in 30 μL methanol/chloroform (1:1) and centrifuged (5 minutes, 16,000*g*, 22°C) before transferring to HPLC vials. A quality control sample was constructed by pooling 3 μL of each sample. Samples (0.5 μL) were injected using a Vanquish Horizon UPLC (Thermo Fisher Scientific) equipped with a Waters ACQUITY Premier CSH (2.1 × 100 mm, 1.7 μm) column operated at 55°C. The analytes were eluted using a flow rate of 400 μL/min and the following composition of eluent A (acetonitrile/water [60:40], 10 mM ammonium formate, 0.1% formic acid) and eluent B (isopropanol/acetonitrile [90:10], 10 mM ammonium formate, 0.1% formic acid): 40% B from 0 to 0.5 minutes, 40%–43% B from 0.5 to 0.7 minutes, 43%–65% B from 0.7 to 0.8 minutes, 65%–70% B from 0.8 to 2.3 minutes, 70%–99% B from 2.3 to 6 minutes, 99% B from 6–6.8 minutes, 99%–40% B from 6.8–7 minutes before equilibration for 3 minutes with the initial conditions. The flow from the UPLC was coupled to a TimsTOF Flex (Bruker) instrument for mass spectrometric analysis. Compounds were annotated in Metaboscape (Bruker) using both an in-built rule-based annotation approach and using the LipidBlast MS2 library (79). Features were removed if their average signal were not greater than 5 times more abundant in the QC samples than blanks (water extraction). The signals were normalized to internal standards in the SPLASH mix before correction for signal drift, blank and QC filtration using Metabolink (80).

The identified lipids were outputted as unique ions as well as ions with multiple adduct forms. Identified adducts with retention times of 0.2 minutes or less apart were combined by summing the raw area counts. The new collapsed adducts were identified by the lipid family name followed by the total number of carbons and double bonds. The QC pool injections were used to calculate the relative standard deviations of the raw area counts for each lipid. Identified lipids with a relative standard deviation of 0.25 or less were uploaded to MetaboAnalyst 6.0 (81) for 1-factor and 2-factor statistical analyses. Missing values were replaced with one-fifth of the lowest positive area count within each feature row. Data lying outside of the interquartile range were removed (*n* = 11 removed features). The data were then quantile normalized, log_10_ transformed, and scaled using mean-centering. One-factor and 2-factor analysis of variance (ANOVAs) were performed with FDR corrections. An α level of 0.05 was considered significant. Data were analyzed, and heatmaps and PCA plots were created using MetaboAnalyst (81).

For the cell lipid analysis, pairwise comparisons were performed on 772 lipid species against either genotype or treatment condition using linear regression models. Lipid concentrations were log_10_ transformed prior to analysis. Lipid species with 3 or more missing values were excluded, while samples with singular missing values were omitted from the analysis for the respective lipid. Regression coefficients and 95% confidence intervals were converted to log_2_(fold change) for interpretation of results. *P* values were corrected for multiple comparisons using the Benjamini-Hochberg method.

### Gene expression.

Total RNA was isolated from approximately 50 mg of pulverized gastrocnemius muscle tissue using TRIzol reagent (Invitrogen, Thermo Fisher Scientific). cDNA was synthesized from 2 μg of isolated RNA (High-Capacity cDNA Reverse Transcription Kit, Thermo Fisher Scientific). qPCR was performed with 6 ng of cDNA in white 384-well plates using PowerUp SYBR Green PCR Master Mix (Applied Biosystems) and the LightCycler 480 II (Roche) instrument. Threshold cycle (C_t_) values were recorded and analyzed using the 2^–ΔΔCt^ method with the expression of *Actb* or *GAPDH* used as a housekeeping gene. Primer sequences used are listed in Table 1.

### Statistics.

Statistical analysis of the data was done using GraphPad Prism 10.1.1 software. Results are presented as mean values ± SEM. Unpaired 2-tailed *t* test, multiple *t* test, 2- or 1-way ANOVA was performed depending on the type of data, followed by Holm-Šídák post hoc analysis unless otherwise stated. Statistical significance was considered when the *P* value was less than 0.05 as follows.

### Study approval.

All murine protocols were approved by the Danish Animal Experiments Inspectorate and performed according to Animal Research: Reporting of In Vivo Experiments (ARRIVE) standards.

### Data availability.

Proteomics data have been deposited at the ProteomeXchange Consortium via the PRIDE partner repository (82) with the dataset identifier PXD077710. Lipidomics data have been deposited at the following repository: https://doi.org/10.6084/m9.figshare.32105305 All deposited data are publicly available as of the date of publication. Values for all data points in graphs are reported in the Supporting Data Values file. Other data will be shared upon reasonable request to the corresponding author.

## Author contributions

TJG designed research studies. KBK, AV, SAJT, FF, JFH, YLS, IB, NRA, ASH, KTM, AMF, SL, NF, and TJG conducted experiments. KBK, AV, SAJT, FF, JFH, IB, NRA, ASH, and TJG acquired data. KBK, AV, SAJT, FF, JFH, YLS, IB, NRA, ASH, ZGH, AMF SL, NF, and TJG analyzed data. MRL, ZGH, SMC, AMF, JTT, SL, and MPG provided reagents. TJG and KBK wrote the manuscript.

## Conflict of interest

The authors have declared that no conflict of interest exists.

## Funding support

- Novo Nordisk Foundation grants NNF21OC0067271 (to TJG), NNF20OC0064744 (to NFJ and JFH), and NNF22OC0079577 (to IB).
- Independent Research Fund Denmark grant 2100-00047B (to TJG).
- Carlsberg Foundation grant CF21-0505 (to TJG).
- Austrian Science Fund (FWF) grants 10.55776/J4822 (to KBK) and 10.55776/J4944 (for IB).
- Barth Syndrome Foundation (to SMC).
- NIH grants R01HL165729 and R01GM15174 (to SMC).
- Danish National Mass Spectrometry Platform for Proteomics and Biomolecular Imaging (www.sdu.dk/PLATO) grant 5229-00012B.

## Supplementary Material

Supplemental data

Unedited blot and gel images

Supporting data values

## Figures and Tables

**Table 1:**
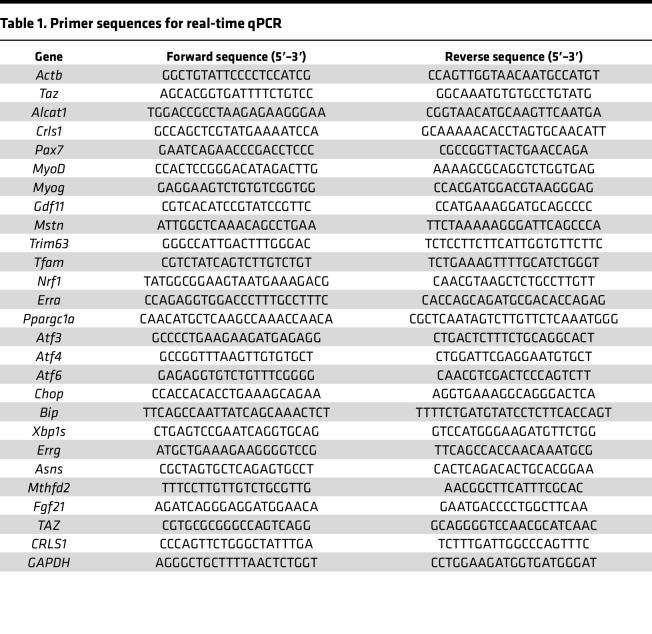
Primer sequences for real-time qPCR

## Version 1 06/09/2026

In-Press Preview

## Version 2 08/10/2026

Electronic publication
