# Supplementary material for: Omega-3 fatty acid supplementation improves skeletal muscle mitochondrial function in a model of Barth syndrome
Source: JCI Insight. 2026 Jun 9;11(15):e196134. doi: 10.1172/jci.insight.196134 (PMC13463623; doi:10.1172/jci.insight.196134)
Supplement: Supplementary file 1 [file jciinsight-11-196134-s041.pdf]

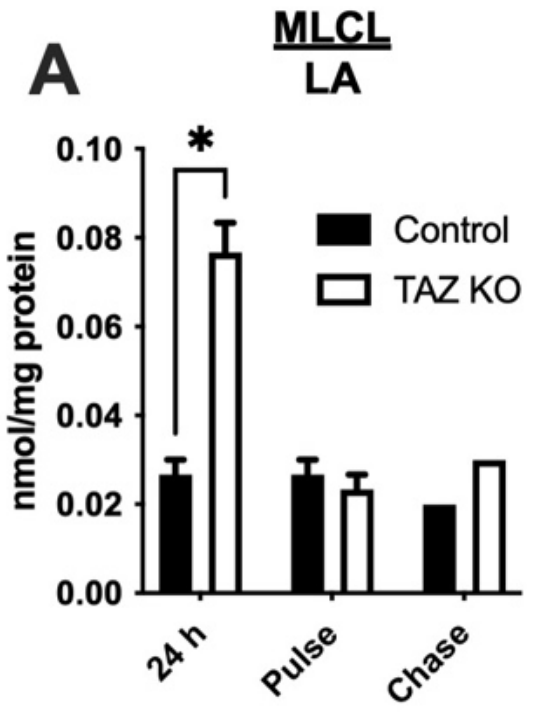

Supplemental Figure 1. Effects of TAZ KO on lipid turnover in HEK293 cells. HEK293 cells were incubated with a mix of fatty acids and 1-[^{14}C]labeled fatty acid for 24 h, 4 h (pulse) or 4 h and then 20 h with only unlabled fatty acid (chase). No MLCL was detected with ARA or DHA labeling. Experiments were performed in triplicate in 2-3 independent assays.

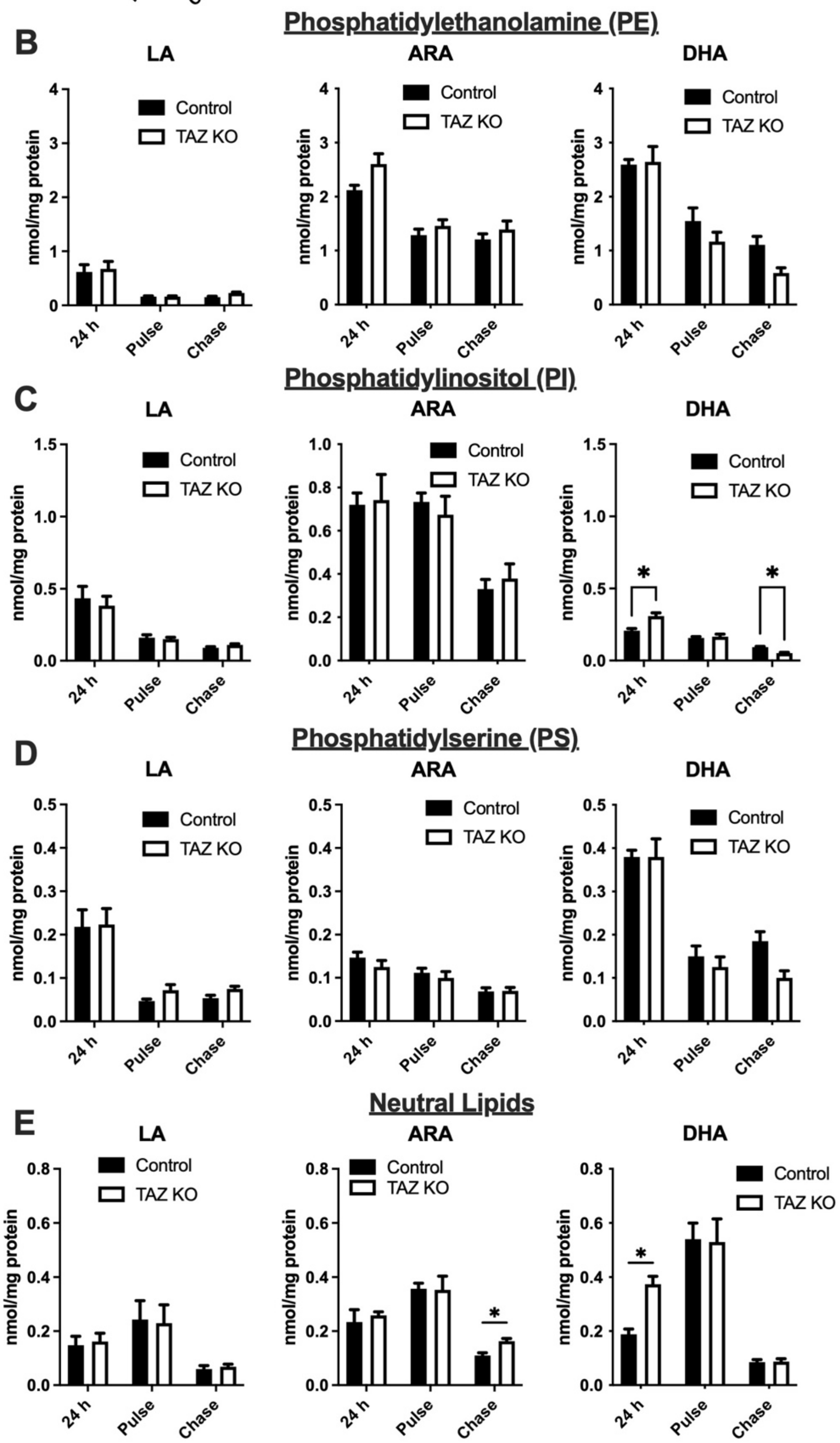

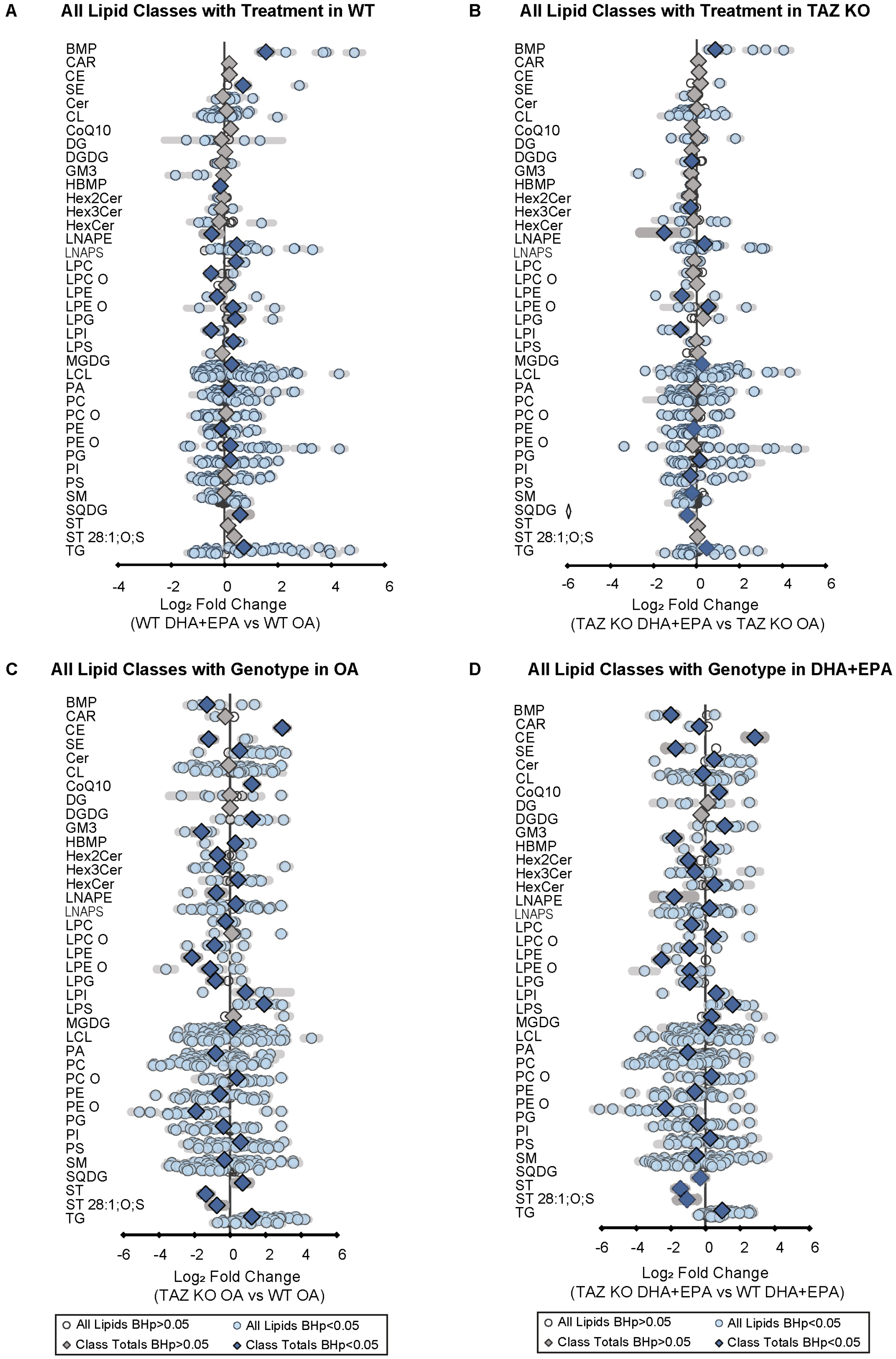
A
All Lipid Classes with Treatment in WT
B
All Lipid Classes with Treatment in TAZ KO
C
All Lipid Classes with Genotype in OA
D
All Lipid Classes with Genotype in DHA+EPA
BMP
CAR
CE
SE
Cer
CL
CoQ10
DG
DGDG
GM3
HBMP
Hex2Cer
Hex3Cer
HexCer
LNAPE
LNAPS
LPC
LPC O
LPE
LPE O
LPG
LPI
LPS
MGDG
LCL
PA
PC
PC O
PE
PE O
PG
PI
PS
SM
SQDG
ST
ST 28:1;O;S
TG
-4 -2 0 2 4 6
Log₂ Fold Change
(WT DHA+EPA vs WT OA)
-6 -4 -2 0 2 4 6
Log₂ Fold Change
(TAZ KO DHA+EPA vs TAZ KO OA)
-6 -4 -2 0 2 4 6
Log₂ Fold Change
(TAZ KO OA vs WT OA)
-6 -4 -2 0 2 4 6
Log₂ Fold Change
(TAZ KO DHA+EPA vs WT DHA+EPA)
All Lipids BHp>0.05
All Lipids BHp<0.05
Class Totals BHp>0.05
Class Totals BHp<0.05

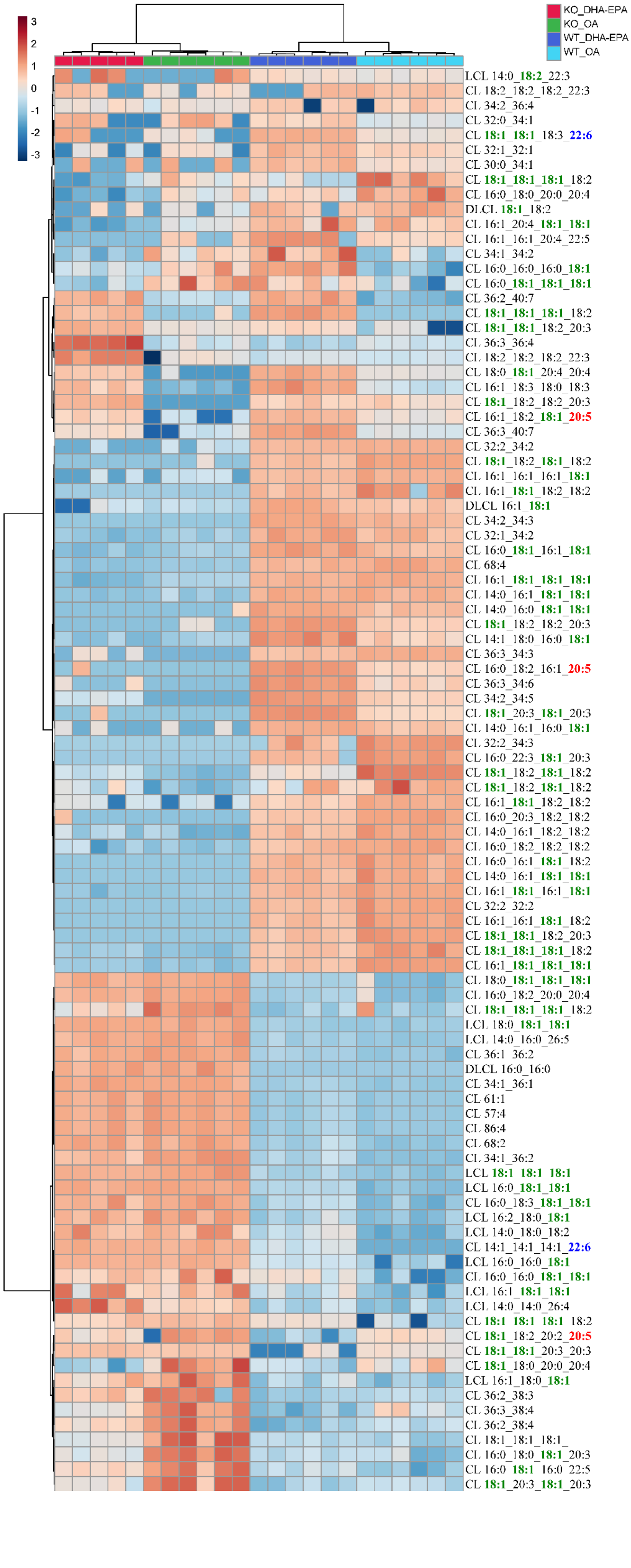

Supplemental Figure 2: Lipidomics analysis of WT and TAZ KO C2C12 cells. Forrest Plots of all lipid classes with (A-B) treatment or (C-D) genotype. (E) Heatmap of all Cardiolipin species. (n=5-6)

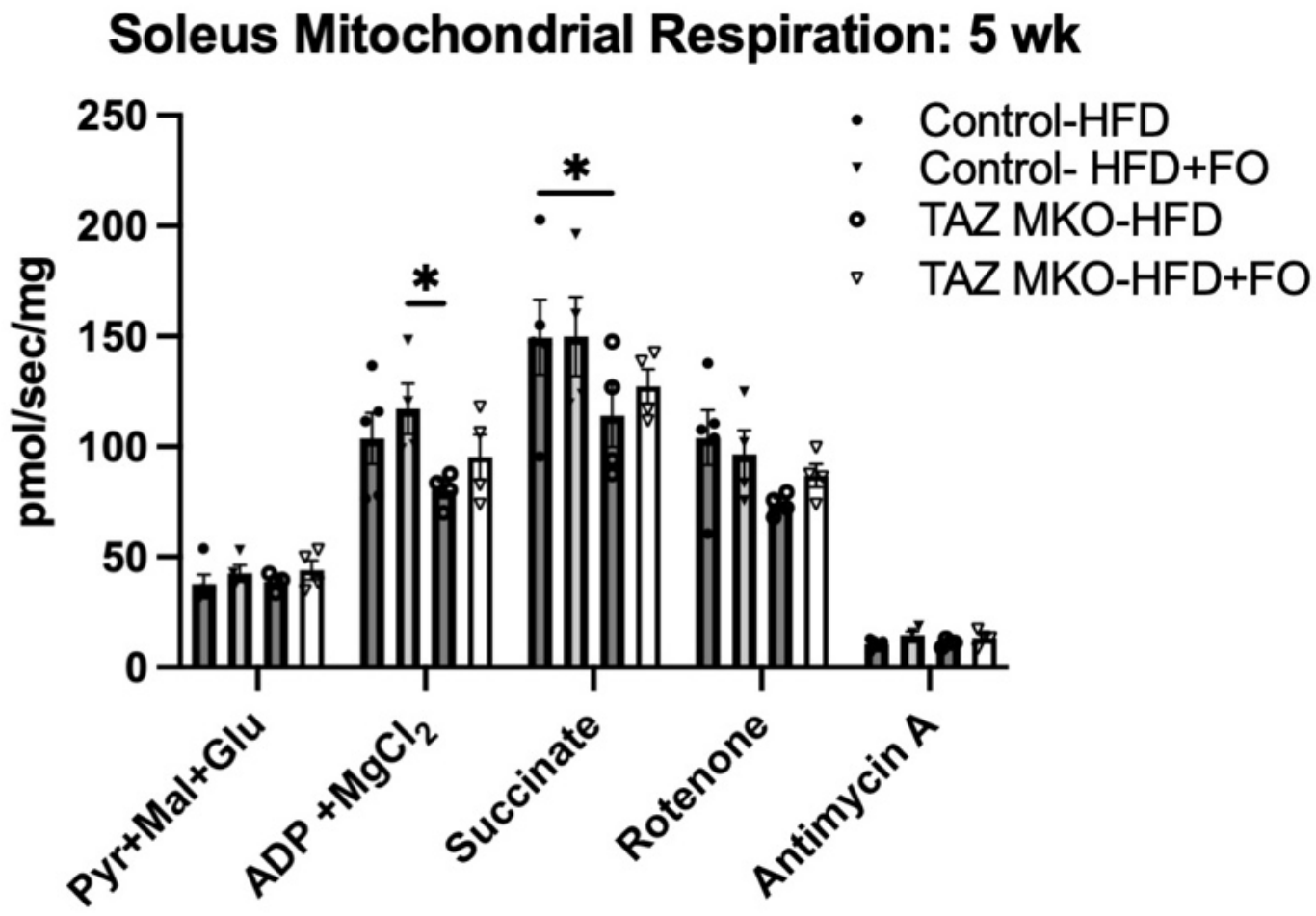

Supplemental Figure 3. *Soleus mitochondrial respiration at an early timepoint.* High resolution respirometry in soleus after 5 weeks of diet feeding (n=4-5). Statistical significance was determined by 2-way ANOVA with Holm-Sidak method for multiple comparison testing where appropriate. *p-value≤0.05

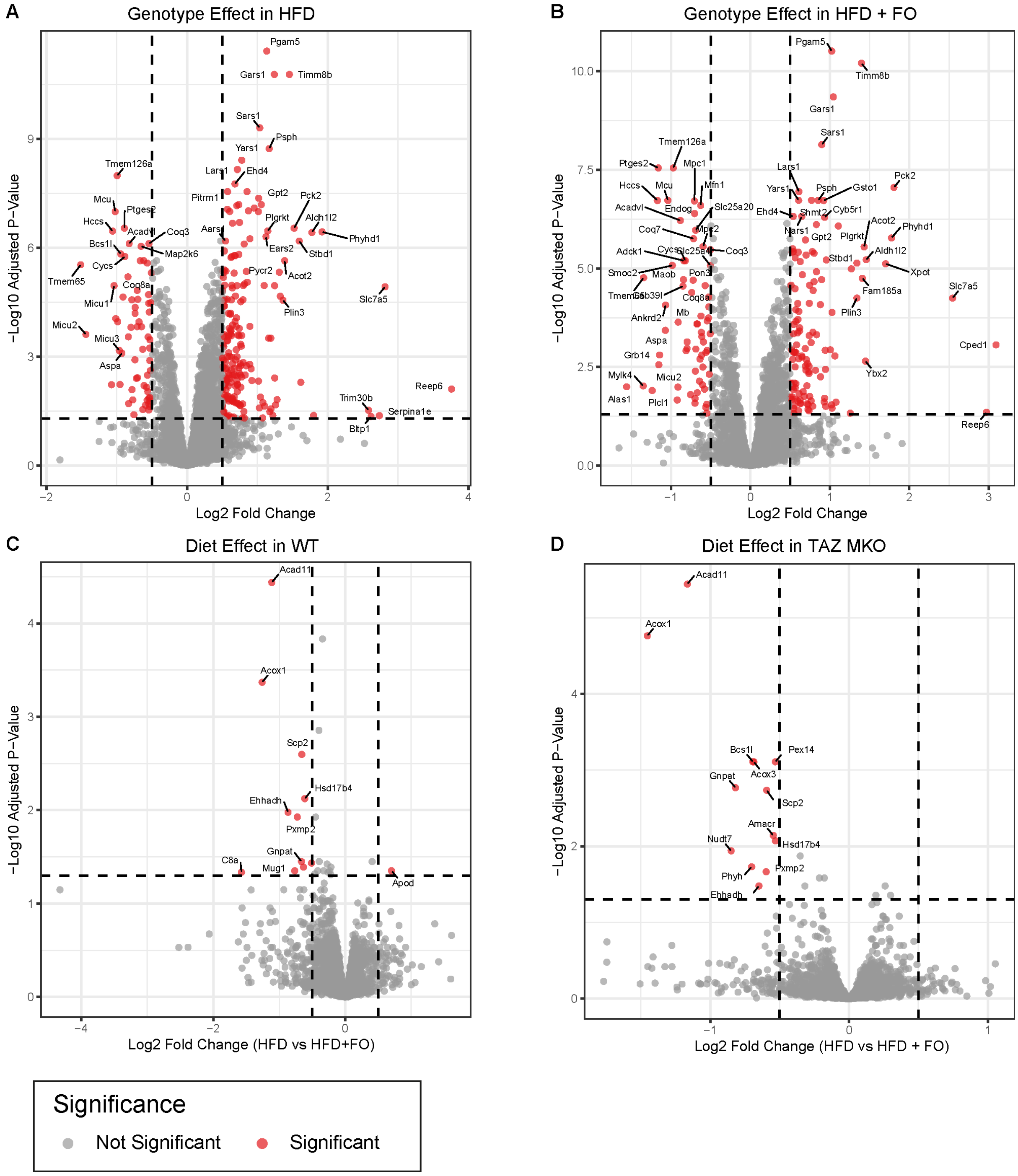

Supplemental Figure 4: Proteomics analysis of Control and TAZ MKO gastrocnemius. Volcano plots showing (A-B) genotype (TAZ MKO vs WT) or (C-D) diet (HFD vs HFD+FO) (n=4-6)

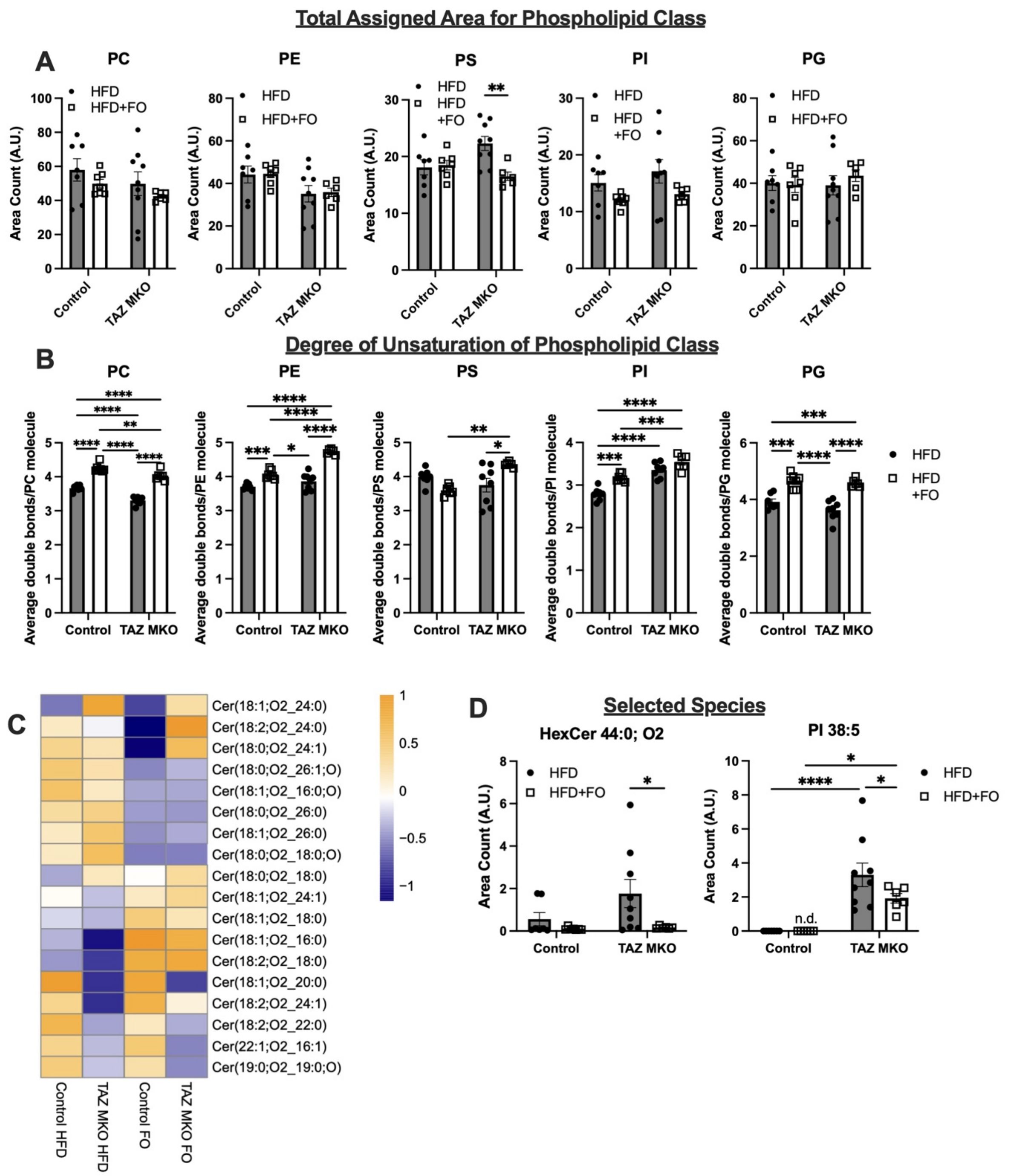

Supplemental Figure 5. Effects of diet and genotype on phospholipids in soleus. **A)** Total assigned area for the phospholipid classes. **B)** Degree of unsaturation of phospholipid acyl chains in soleus (n=6-9). **C)** Heatmap of ceramides. **D)** Selected species with diet-genotype interactions. (n=6-9)
