# Supplementary material for: Omega-3 fatty acid supplementation improves skeletal muscle mitochondrial function in a model of Barth syndrome
Source: JCI Insight. 2026 Jun 9;11(15):e196134. doi: 10.1172/jci.insight.196134 (PMC13463623; doi:10.1172/jci.insight.196134)

Uncropped WB

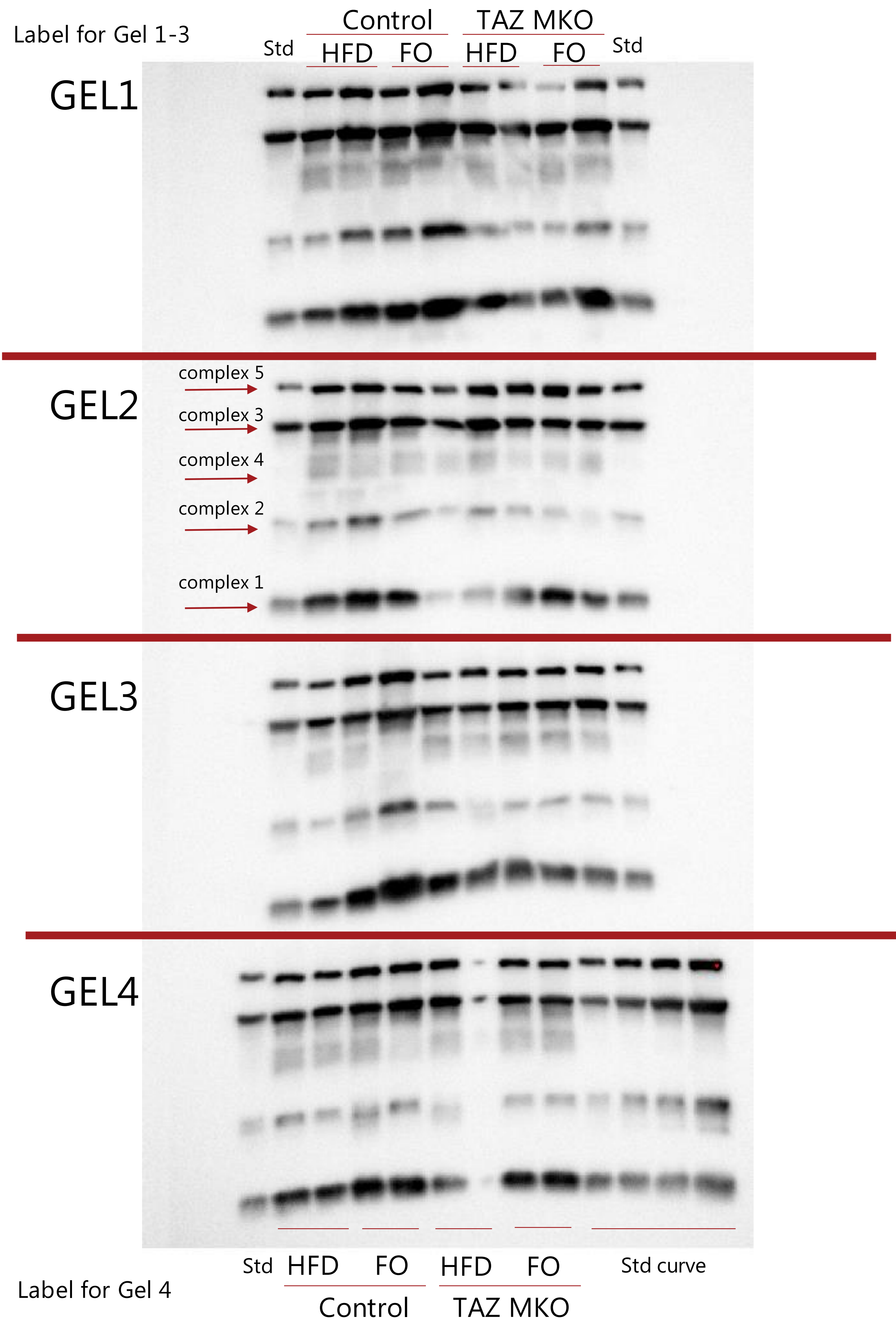

Supplement: Supplementary file 2 [file jciinsight-11-196134-s042.pdf]
